# Chronic pulmonary bacterial infection facilitates breast cancer lung metastasis by recruiting tumor-promoting MHCII^hi^ neutrophils

**DOI:** 10.1038/s41392-023-01542-0

**Published:** 2023-08-11

**Authors:** Teng Ma, Yu Tang, Taolin Wang, Yang Yang, Yige Zhang, Ruihuan Wang, Yongxin Zhang, Yi Li, Mingbo Wu, Miao Tang, Xueli Hu, Chaoyu Zou, Yuan Ren, Huan Liu, Qianhua Zhang, Heyue Li, Min Wu, Jing Li, Xikun Zhou

**Affiliations:** 1 Department of Biotherapy, Cancer Center and State Key Laboratory of Biotherapy, West China Hospital, Sichuan University, 610041 Chengdu, China; 2 Department of Breast Surgery, Sichuan Provincial People’s Hospital, University of Electronic Science and Technology of China, 610072 Chengdu, China; 3 State Key Laboratory of Oral Diseases, National Clinical Research Center for Oral Diseases, Research Unit of Oral Carcinogenesis and Management, Chinese Academy of Medical Sciences, West China Hospital of Stomatology, Sichuan University, Chengdu, China; 4 Drug Discovery Center, Wenzhou Institute, University of Chinese Academy of Sciences, 325001 Wenzhou, China

**Keywords:** Breast cancer, Metastasis, Inflammation

## Abstract

Breast cancer can metastasize to various organs, including the lungs. The immune microenvironment of the organs to be metastasized plays a crucial role in the metastasis of breast cancer. Infection with pathogens such as viruses and bacteria can alter the immune status of the lung. However, the effect of chronic inflammation caused by bacteria on the formation of a premetastatic niche within the lung is unclear, and the contribution of specific immune mediators to tumor metastasis also remains largely undetermined. Here, we used a mouse model revealing that chronic pulmonary bacterial infection augmented breast cancer lung metastasis by recruiting a distinct subtype of tumor-infiltrating MHCII^hi^ neutrophils into the lung, which exhibit cancer-promoting properties. Functionally, MHCII^hi^ neutrophils enhanced the lung metastasis of breast cancer in a cell-intrinsic manner. Furthermore, we identified CCL2 from lung tissues as an important environmental signal to recruit and maintain MHCII^hi^ neutrophils. Our findings clearly link bacterial-immune crosstalk to breast cancer lung metastasis and define MHCII^hi^ neutrophils as the principal mediator between chronic infection and tumor metastasis.

## Introduction

Breast cancer is a highly malignant tumor type, and lung metastasis is an important cause of death in breast cancer patients. The occurrence of breast cancer lung metastasis is closely related to chronic inflammation caused by several factors, including stress, obesity, and smoking.^1–3^ Patients with breast cancer and other kinds of tumors are plagued by tumor cells and often suffer from immune injury caused by radiotherapy and chemotherapy. The weakened immune defense ability of tumor patients leads to their susceptibility to chronic pulmonary infection caused by pathogenic bacteria. Therefore, the relationship between the chronic inflammation caused by pathogenic bacteria and metastasis of breast cancer is of important research.

There is a strong epidemiological link between bacterial infections and cancer.^4^ Specifically, there are connections between chronic *Salmonella Typhi* (*S. Typhi*) infections and gallbladder carcinoma^5^ and between *Helicobacter pylori* (*H. pylori*) infections and gastric cancer.^6^ Bacteria can impact multistep processes of cancer formation and development by directly influencing cancer cells through bacterial effector proteins or changing the immune microenvironment.^5,7^ Considerable attention has been given to the effects of bacteria on cancer, whereas relatively little interest has focused on the effects of chronic inflammation caused by bacteria on cancer. Moreover, the role of distal (lung) pathogenic bacteria in breast cancer has not been elucidated.

It has been reported that the immune status of distant metastatic organs is important for tumor cell colonization and metastasis, suggesting that there may be a strong correlation between pulmonary bacterial infection and lung metastasis of breast cancer. Inflammatory conditions induced by exogenous pathogens and endogenous factors can alter the infiltration of immune cells, including neutrophils, which is associated with clinical outcome in breast cancer patients. Neutrophils are the most abundant leukocytes (50–70%) in human peripheral blood and the first line of cellular defense of host immune systems against microbial infections.^8^ Recent research progress on the role of neutrophils in the tumor microenvironment has revealed that neutrophils possess both pro- and anti-tumor properties.^9^ However, the specific phenotypes and mechanisms of these properties remain unclear. Numerous studies have demonstrated that neutrophils with distinct protumor phenotypic characteristics are associated with a poor prognosis in patients with a variety of solid tumors.^10^ In contrast, other studies have shown that neutrophils are biomarkers of improved survival rates in patients with various cancer types.^11,12^ Thus, these observations suggest that neutrophils are heterogeneous and dynamic cells; however, how these features enable neutrophils to adapt to their tissue microenvironment to meet functional demands is a major challenge in the field.

Here, we established a widely used chronic pulmonary infection (CI) mouse models by transtracheal injection of agar bead-embedded *Pseudomonas aeruginosa* (*P. aeruginosa*), which is one of the most common pathogens and causes acute and chronic respiratory infections. Using cytometry by time of flight (CyTOF) to characterize the immune cell status of chronically infected lung tissues, we identified a distinct population of MHCII^hi^ neutrophils. The MHCII^hi^ neutrophils recruited by CI-derived CCL2 exhibited cancer-promoting functions both in vitro and in vivo. These results indicate that MHCII^hi^ neutrophils may be a potential therapeutic target for lung metastasis to improve the management of patients with late-stage cancer.

## Results

### Chronic pulmonary infection enhances breast cancer lung metastasis

**Fig. 1:**
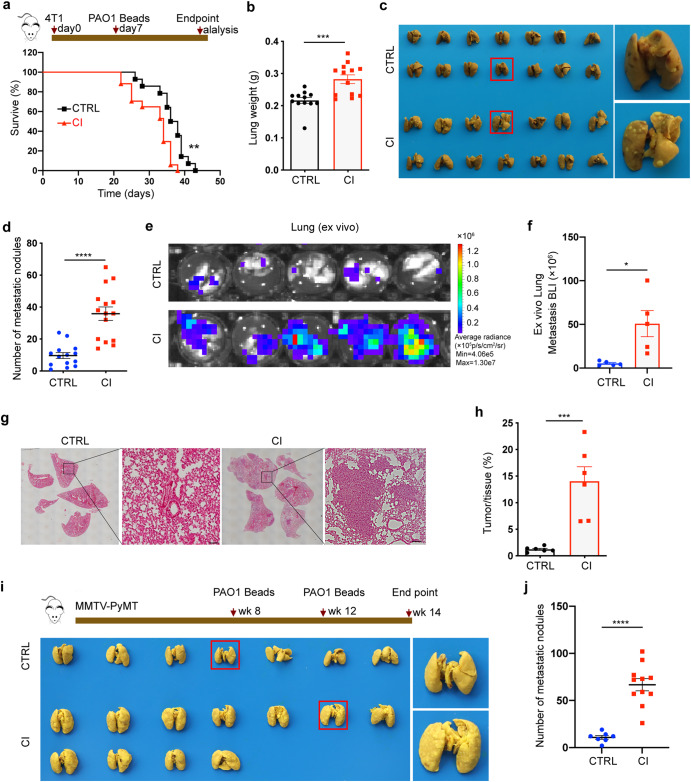
Chronic pulmonary *P. aeruginosa* infection promotes breast cancer lung metastasis. **a** Upper panel: Schematic diagram of spontaneous metastasis. Mouse mammary carcinoma 4T1 cells (2 × 10^5^) were orthotopically implanted into mouse MFPs followed by tracheal injection of PBS or PAO1-Beads on day 7 after tumor bearing. Lower panel: Kaplan–Meier analysis of lung metastasis survival in PBS (*n* = 17) or PAO1-bead-treated (*n* = 14) mice [log-rank (Mantel–Cox test). PAO1-Beads agar bead-embedded PAO1, MFP mammary fat pad. **b** A mouse model was constructed as described above, and the mice were sacrificed at the endpoint. Weight of lungs excised from mice in different treatment groups at day 21 (*n* = 12–13 mice/group). **c**, **d** Lung images (**c**) and numbers of lung metastasis nodules (**d**) from CTRL (control) group and CI (chronic infection) group mice (*n* = 14–15 mice/group). **e**, **f** Representative images of ex vivo metastatic lungs (**e**) and ex vivo quantification of lung metastasis (**f**) from 4T1-luci-bearing mice with or without chronic pulmonary PAO1 infection (*n* = 5 mice/group). **g**, **h** Representative H&E pictures (**g**) and corresponding quantification (**h**) of lung tissues are shown to determine the tumor burden (*n* = 6 mice/group). Scale bar, 25 μm **I**, **j** Bright-field imaging (**i**) and number of metastatic nodules (**j**) in the lungs of MMTV-PyMT mice from the CTRL group (*n* = 7) and CI group at the endpoint (14 weeks of age) (*n* = 11). Scale bar, 50 μm. All data are presented as the mean ± SEM. Statistical significance was calculated using an unpaired *t* test. ****P < 0.0001; ***P < 0.001; **P < 0.01; *P < 0.05

To assess the association between chronic pulmonary bacterial infection and the development of breast cancer lung metastasis, spontaneous metastasis mouse models were first established by orthotopic injection of 4T1 mouse mammary cancer cells. Then, these mice received an intratracheal instillation of agar bead-embedded PAO1 (*P. aeruginosa* reference strain) on day 7 after tumor bearing, similar to our previous report^13^ (Fig. 1a). Notably, the mortality rate of tumor-bearing mice chronically infected (CI) with PAO1 significantly increased compared to that of the control group (CTRL) (Fig. 1a). The tumor weight and volume did not differ statistically between the two groups (Supplementary Fig. 1a–c). Previous studies have shown that breast cancer commonly metastasizes to the brain, bone, liver and lung.^14^ Metastasis is the major cause of death among breast cancer patients.^15^ We therefore examined the role of chronic infection in tumor cell metastasis with our mouse models in vivo. On day 21, both groups had enlarged spleens, and there was no significant difference between these two groups (Supplementary Fig. 1d), and no metastatic foci were observed in the spleen. Intriguingly, the lung weight of the CI group was significantly higher than that of the CTRL group (Fig. 1b). Lungs were then fixed in Bouin’s solution, and lung metastasis nodules were counted. The statistical results showed that the number of lung metastases in the CI group was much greater than that in the CTRL group (Fig. 1c, d). This result was also confirmed by ex vivo bioluminescence imaging (Fig. 1e, f). Compared to the CTRL mice, CI tumor-bearing mice exhibited enhanced tumor burdens on day 21, as shown by hematoxylin-eosin staining (H&E) (Fig. 1g, h). Additionally, we performed the same experiment in spontaneous breast cancer MMTV-PyMT model mice. Similarly, chronic pulmonary infection caused by PAO1 increased lung weight of MMTV-PyMT mice (Supplementary Fig. 1e) and enhanced breast cancer lung metastasis (Fig. 1i, j) without affecting orthotopic tumor weight (Supplementary Fig. 1f, g).

Although 4T1 mammary carcinoma cell line is a highly tumorigenic and invasive cell line and can spontaneously metastasize from the mammary gland to the lung, our results indicated that chronic bacterial infection accelerated this process. To confirm whether the alteration of the premetastatic niche caused by chronic pulmonary bacterial infection promotes breast cancer lung metastasis from another aspect, we chronically infected the lungs of mice with PAO1 beads before orthotopic 4T1 tumor implantation. The results showed that the early formation of a metastatic niche caused by chronic pulmonary bacterial infection could promote lung metastasis of breast cancer in the mouse model (Supplementary Fig. 2a, b). The effects of pathogenic microorganisms during acute and chronic infections on hosts or tumors are remarkably different. To determine whether acute bacterial infection has the same effects on promoting tumor cell metastasis, we constructed an acute pulmonary infection mouse model with PAO1 (AI group), as in our previous study,^13^ which is similar to those described above (Supplementary Fig. 2c). In contrast, the AI group exhibited fewer lung metastasis nodules than the CTRL group (Supplementary Fig. 2c, d). Collectively, these results demonstrate that chronic and acute bacterial infections in the mouse lung play different roles in breast cancer lung metastasis.

A variety of bacterial infections can cause chronic pulmonary infections. *Staphylococcus aureus* (*S. aureus*) is also one of the most commonly-detected gram-positive bacteria in the clinic and can cause mild to fatal infections in almost all organs, including the skin and lungs. We further tested whether *S. aureus*-induced chronic infection can also promote lung metastasis of breast cancer. Thus, we established a 4T1-bearing mice model followed by chronic pulmonary *S. aureus* infection as described above. This result was consistent with the results of our models or tests (Supplementary Fig. 2e, f).

### Chronic pulmonary infection affects the status of immune cells in lung tissues

**Fig. 2:**
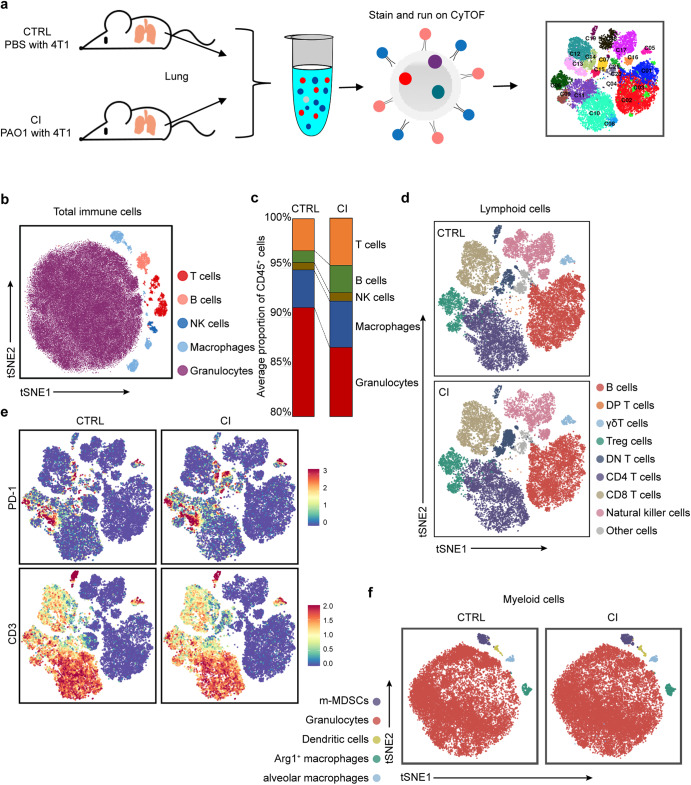
CyTOF profiling analyzes immune cells in chronically infected lung tissues. **a** Study design: Lungs were collected from 4T1-bearing mice with or without chronic PAO1 infection at the endpoint (21 days) and were analyzed by CyTOF. Unique immune subsets were identified by CyTOF analysis (*n* = 2 mice/group). **b** Two-dimensional t-SNE illustration of the merged CyTOF data from the lungs of both the CTRL group and CI group. **c** Frequency of immune lineages based on summation of Phenograph metaclusters. Composition of the CD45^+^ compartment showing average frequencies of major immune lineages in the lung tissue from the CTRL group and CI group (*n* = 2 mice/group). **d** viSNE analysis of lymphoid cells colored and labeled by Phenograph metacluster for the two groups. **e** Normalized expression of PD-1 in lung CD3^+^ cells shown by the viSNE plot for the CTRL and CI groups. **f** viSNE analysis of myeloid cells colored and labeled by Phenograph metacluster for the two groups

Based on the assumption that chronic pulmonary *P. aeruginosa* infection could alter the immune environment of the lung to impact the process of breast cancer-derived lung metastases, we next examined the difference in immune subsets in the lung tissues between the CI group and the CTRL group and performed single-cell mass cytometry (CyTOF) using the whole lungs of the two groups on day 21 (Fig. 2a). A panel consisting of 41 immune protein markers (Supplementary Table 1) was used to delineate the major immune cell types and map the high-dimensional landscapes of multiple immune checkpoints. We generated a single-cell immune atlas with healthy and infected tumor-bearing mouse lung tissues for several clusters, including T cells, B cells, natural killer (NK) cells, and myeloid cells, using well-characterized cell markers presented as two-dimensional (2D) t-SNE plots (Fig. 2b). The percentages of each immune lineage within the gated CD45^+^ population were manually gated using FlowJo into two groups. Notably, myeloid cells accounted for the majority of total immune cells in both groups according to the CyTOF results. Unsurprisingly, the proportion of all these cells in the CI group was slightly smaller than that in the CTRL group (Fig. 2c).

To analyze cell subpopulations accurately, lymphoid markers were extracted and clustered into B cells, T-cell subpopulations, and NK cells. There was no appreciable difference in those cell subtypes between these two groups (Fig. 2d). Remarkably, compared to the CTRL group, CD3^+^ T cells of the CI group in the lungs expressed higher levels of PD-1, a key immunosuppressive factor in the tumor microenvironment, which was also validated by flow cytometry (Fig. 2e and Supplementary Fig. 3a, b). In addition, we extracted myeloid markers and then clustered them into granulocytes (neutrophils), alveolar macrophages, Arg^+^ macrophages (M2 macrophages), dendritic cells (DCs) and m-MDSCs (Fig. 2f). The proportion of neutrophils decreased according to the CyTOF results, while the validation results by flow cytometry showed no significant difference (Supplementary Fig. 3c–e). Within the myeloid lineages, except for neutrophils, we found a significant increase in both the number and proportion of lung Arg^+^ macrophages (M2 macrophages) in CI mice versus CTRL mice using CyTOF (Fig. 2f) and flow cytometry (Supplementary Fig. 3f, g). Although CI group mice exhibited increased proportions of several immune subtypes that promote tumor progression and immune evasion, the total percentage of those immune cells among CD45^+^ cells was quite low. Therefore, we next focused our investigation on neutrophils.

### MHCII^hi^ neutrophil numbers are increased in the lung tissues of chronic pulmonary infections

**Fig. 3:**
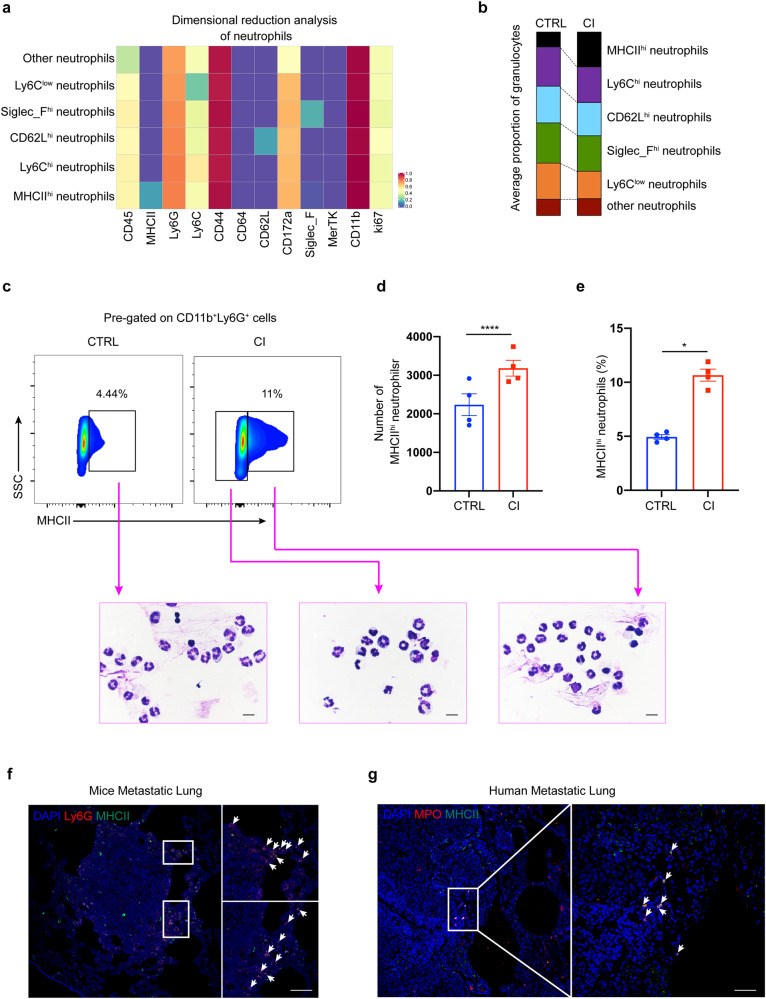
Identification of MHCII^hi^ neutrophils in the lung tissues of breast cancer-bearing mice. **a** Heatmap showing the differential expression of 12 neutrophil-related immune markers in the two groups. The immune subsets that represent these nodes based on their marker expression are noted. **b** The proportion of histogram showing the frequency of neutrophil subsets. **c** Flow cytometry-based detection (upper panel) of MHCII^hi^ or MHCII^low^ neutrophils from the lung tissue of tumor-bearing mice with (lower right) or without chronic PAO1 infection (lower left). Plots are shown for gated live CD45^+^CD11b^+^Ly6G^+^ cells. Representative cytospin images (bottom) are from FACS (fluorescence-activated cell sorting)-sorted populations and Swiss-Giemsa staining. Scale bar, 10 μm. **d**, **e** Quantification (**d**) and frequency (**e**) of MHCII^hi^ neutrophils in (**c**), *n* = 4 mice/group. **f** Confocal immunofluorescent images of lung tissues from tumor-bearing mice with chronic PAO1 infection. Blue: DAPI; red: Ly6G; green: MHCII. Scale bar, 50 μm. **g** Confocal immunofluorescent images of lung tissues from patients with breast cancer. Blue: DAPI; red: MPO; green: MHCII. Scale bar, 50 μm. All data are presented as the mean ± SEM. Statistical significance was calculated using an unpaired *t* test. ****P < 0.0001; *P < 0.05

Neutrophils have been shown to promote or inhibit lung metastasis of breast cancer in different environmental conditions.^2,16^ Recent studies have demonstrated that neutrophils are heterogeneous,^17,18^ but the classes and functions of neutrophil subtypes are much less studied than those of other immune cells, such as macrophages and T cells. Cluster analysis of neutrophils indicated that there were at least six populations of neutrophil subtypes in the lung tissues of our mouse models, including MHCII^hi^ neutrophils, Ly6C^hi^ neutrophils, CD62L^hi^ neutrophils, Siglec_F^hi^ neutrophils, Ly6C^low^ neutrophils and other neutrophil subsets (Fig. 3a). Notably, the results of CyTOF analysis revealed that MHCII^hi^ neutrophils were present at a higher frequency in the lungs of the CI group than in the CTRL group. In contrast, the other subtypes of neutrophils were equally represented in these two groups (Fig. 3b). Similar results were obtained in flow cytometry experiments (Fig. 3c–e). However, we did not observe a significant change in the proportion of total neutrophils or MHCII^hi^ neutrophils in the peripheral blood (Supplementary Fig. 4a–c). In addition, we analyzed the proportion of MHCII^hi^ neutrophils in the lung tissue of MMTV-PyMT mice after chronic pulmonary PAO1 infection using flow cytometry. As expected, the results were consistent with those in 4T1 tumor-bearing mice (Supplementary Fig. 5a–c). Furthermore, only chronic PAO1 infection promoted the accumulation of MHCII^hi^ neutrophils in the lungs of tumor-free mice (Supplementary Fig. 5d, e). These results suggest that there was a significantly higher level of MHCII^hi^ neutrophil recruitment to the lungs of the CI group due to chronic pulmonary *P. aeruginosa* infection. To investigate the relationship between MHCII^hi^ neutrophils and tumor development, we first analyzed the location of MHCII^hi^ neutrophils in the lungs of our model mice. Surprisingly, immunohistochemical staining showed that MHCII^hi^ cells clustered around and within tumor nodules (Supplementary Fig. 5f). Immunofluorescence staining further confirmed that MHCII^hi^ neutrophils appeared in the periphery of tumor nodules (Fig. 3f). To verify whether MHCII^hi^ neutrophils are specific for mice or are also present in patients, lung tissue samples were collected from patients with breast cancer lung metastases. Immunofluorescence assays of patient samples also confirmed that MHCII^hi^ neutrophils were distributed in the periphery and inside the tumor. The presence of MHCII^hi^ neutrophils was detected in lung tissue samples collected from 9 of 11 patients (Fig. 3g and Supplementary Table 2). These results indicate that MHCII^hi^ neutrophils may play a role in breast cancer metastasis to lungs.

### MHCII^hi^ neutrophils possess distinct tumor-promoting transcriptomic signatures

**Fig. 4:**
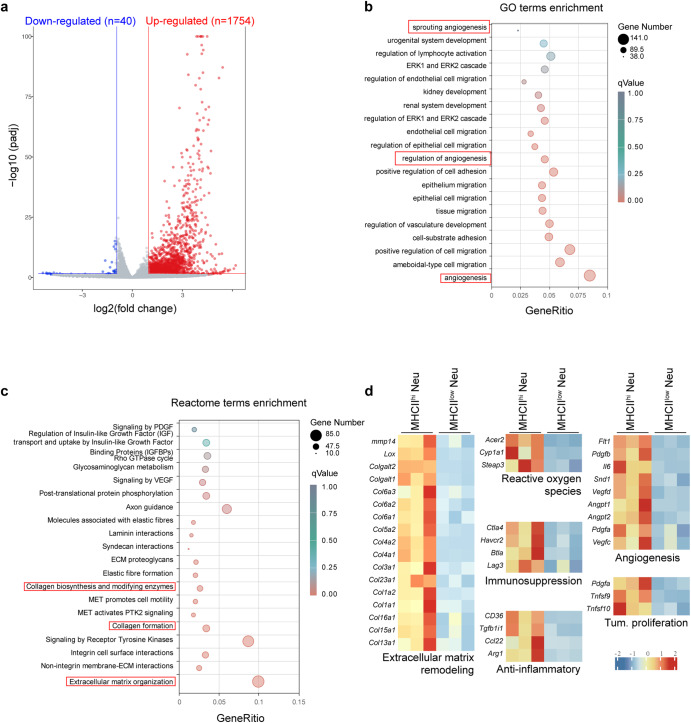
Transcriptome analysis of MHCII^hi^ and MHCII^low^ neutrophils isolated from the lungs of tumor-bearing mice with chronic PAO1 infection. **a** Volcano plot showing differential gene expression between MHCII^hi^ and MHCII^low^ neutrophils in the lungs of 4T1-bearing mice with chronic PAO1 infection. Genes with a fold change higher than 2 and a *P* value of <0.05 are highlighted in blue and red, denoting down- and upregulated genes, respectively, in MHCII^hi^ and MHCII^low^ neutrophils. **b** GO enrichment in MHCII^hi^ and MHCII^low^ neutrophils in the lungs of 4T1-bearing mice with chronic PAO1 infection. **c** Reactome pathway analysis of the enriched genes identified from MHCII^hi^ and MHCII^low^ neutrophils in the lungs of 4T1-bearing mice with chronic PAO1 infection. **d** Average expression levels of genes involved in extracellular matrix remodeling, reactive oxygen species, immunosuppression, anti-inflammation, angiogenesis, and tumor proliferation in MHCII^hi^ and MHCII^low^ neutrophils in the lungs of 4T1-bearing mice with chronic PAO1 infection

To further understand the roles of neutrophil subpopulations, we specifically asked whether MHCII^hi^ neutrophils exhibit tumor-promoting properties. To this end, we performed RNA sequencing (RNA-seq) on MHCII^hi^ neutrophils and MHCII^low^ neutrophils isolated from the lungs of tumor-bearing mice. We identified 1754 and 40 genes that were significantly upregulated and downregulated in MHCII^hi^ neutrophils, respectively, with a fold change higher than 2 and a *P* value less than 0.05 (Fig. 4a). These differentially expressed genes were analyzed using Gene Ontology (GO) and Reactome functional analysis tools (Fig. 4b, c). Multiple tumor-associated pathways were significantly enriched. Notably, the upregulation of angiogenesis-related pathways in the GO database suggested that MHCII^hi^ neutrophils might promote angiogenesis to support tumor cell growth. The Reactome database revealed the extracellular matrix remodeling-rated pathways as one of the dramatically changed pathways. We further examined the differentially expressed genes. We found that a number of genes associated with tumor-promoting processes were highly expressed in MHCII^hi^ neutrophils (Fig. 4d), including extracellular matrix remodeling (Lox, Mmp14, and collagens), reactive oxygen species (Acer2, Cyp1a1, and Steap3), suppression of T-cell responses (Ctla4, Btla, Havcr2 and Lag3), anti-inflammatory genes (CD36, Tgfb1i1, Ccl22, and Arg1), angiogenesis (Il-6, Flt1, and Vegfc), and tumor cell proliferation/growth (Pdgfa, Tnfsf9, and Tnfsf10). These results suggest that MHCII^hi^ neutrophils possibly remodel the lung microenvironment to promote tumor cell motility and thus facilitate metastatic colonization.

To explore whether the tumor-promoting phenotype of MHCII^hi^ neutrophils was directly affected by chronic bacterial infection, we isolated MHCII^hi^ neutrophils from the lungs of both CI tumor-bearing mice and CTRL tumor-bearing mice. Then, we compared the transcriptomes of the MHCII^hi^ neutrophils from these two different sources and found similar transcription characteristics (Supplementary Fig. 6a). Furthermore, the difference in tumor-related genes between the two neutrophil groups was not apparent (Supplementary Fig. 6b). The above results indicate that this kind of cells is indirectly influenced by bacterial infection.

The neutrophils in the lungs of tumor-bearing mice are continuously replenished by circulating cells from the peripheral blood. Therefore, we verified whether MHCII^hi^ neutrophils were influenced by tissue-specific factors in the lung microenvironment. Thus, we sorted CD45^+^CD11b^+^Ly6G^+^MHCII^hi^ neutrophils from the blood of tumor-bearing mice and performed RNA-seq. PCA showed a remarkable separation between MHCII^hi^ neutrophils from the blood and those from the lungs of tumor-bearing mice (Supplementary Fig. 7a), suggesting that these two neutrophil groups have distinct cellular characteristics. Volcano plots of RNA-seq data revealed that MHCII^high^ neutrophils in the blood substantially diverged from the cells in the lung (3989 differentially expressed genes; Supplementary Fig. 7b). Multiple pathways associated with cancer development were enriched using the GO database and Reactome functional analysis tool (Supplementary Fig. 7c, d). MHCII^hi^ neutrophils in the lung compared to those in the blood selectively upregulated the expression of genes associated with tumor-promoting processes (Supplementary Fig. 7e). These results indicated that the tumor-promoting phenotypes of MHCII^hi^ neutrophils occur following their migration into lung tissues.

### MHCII^hi^ neutrophils exhibit cancer-promoting functions both in vitro and in vivo

**Fig. 5:**
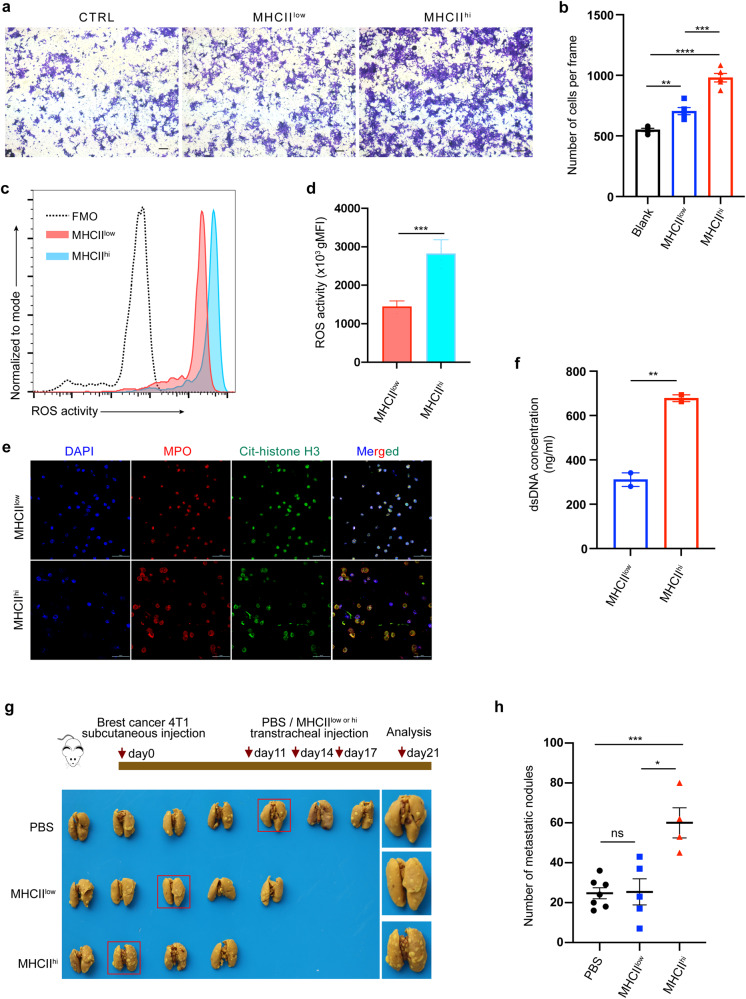
MHCII^hi^ neutrophils show tumor-promoting functions both in vitro and in vivo. **a**, **b** Representative images (**a**) displaying the effects of MHCII^hi^ and MHCII^low^ neutrophils on 4T1 cell migration evaluated using Transwell assays. 4T1 cells were added to the upper chamber, MHCII^hi^ or MHCII^low^ neutrophils were added to the bottom chamber (8 μm pores), and the cells that transmigrated into the lower chamber were counted (**b**). *n* = 3. Scale bar, 200 μm. **c**, **d** Representative histogram (**c**) and quantification of the geometric mean fluorescence intensity (gMFI) (**d**) of ROS activity, measured by rhodamine 123 fluorescence (oxidized dihydrorhodamine 123) using flow cytometry, in MHCII^hi^ and MHCII^low^ neutrophils sorted by FACS (*n* = 4 mice/group). **e** Representative images of LPS-triggered NET formation in vitro. MHCII^hi^ and MHCII^low^ neutrophils sorted by FACS were treated with 100 μg/mg LPS for 3 h and then stained with DAPI (blue), anti-H3cit (green) and MPO (red) for NET formation identification in vitro. Experiments were repeated 3 times, and *n* = 3 replicates in each experiment. Scale bar, 50 μm. **f** Concentration of dsDNA (cell-free DNA) in cultured supernatants from MHCII^hi^ or MHCII^low^ neutrophils treated with 100 μg/mg LPS for 3 h. **g** Bright-field imaging of metastatic nodules in the lungs of tumor-bearing mice infused with PBS, MHCII^hi^ or MHCII^low^ neutrophils for the indicated times. The control PBS, MHCII^hi^ or MHCII^low^ neutrophils were instilled into mice via the trachea on the 11th, 14th, and 17th days after 4T1 cell inoculation (*n* = 4–7 mice/group). **h** Number of metastatic nodules in the lungs derived from (**g**). All data are presented as the mean ± SEM. Statistical values were calculated using one-way ANOVA (**b**, **h**) or an unpaired *t* test (**d**, **f**). ns not significant; ****P < 0.0001; ***P < 0.001; **P < 0.01; *P < 0.05

To evaluate the function of MHCII^hi^ neutrophils compared to MHCII^low^ neutrophils, neutrophils were isolated from tumor-bearing mice by flow sorting. We first performed a Transwell assay to investigate the effect of MHCII^hi^ and MHCII^low^ neutrophils on tumor cell migration (Fig. 5a). Our results show that both MHCII^hi^ and MHCII^low^ neutrophils can promote tumor migration (Fig. 5b). Compared to MHCII^low^ neutrophils, MHCII^hi^ neutrophils had a stronger effect on tumor migration (Fig. 5b). However, there was no significant difference between the roles of MHCII^hi^ and MHCII^low^ neutrophils in tumor invasion, and compared with the blank control, both kinds of neutrophils promoted tumor invasion in Transwell chamber experiments (Supplementary Fig. 8a, b). Furthermore, our neutrophil RNA-seq data revealed that MHCII^hi^ neutrophils highly expressed ROS-related genes (Fig. 4c). As reactive oxygen species (ROS) can drive diverse protumorigenic inflammatory reactions,^19^ we detected the intracellular ROS production capacity of MHCII^low^ and MHCII^hi^ neutrophils by flow cytometry. Although both neutrophil types can produce ROS, MHCII^hi^ neutrophils generate more ROS than MHCII^low^ neutrophils (Fig. 5c, d). Although the neutrophil transcriptome results implied that compared to MHCII^low^ neutrophils, MHCII^hi^ neutrophils have active tumor pro-proliferative roles (Fig. 4d), we did not find a significant difference between the two neutrophil types in in vitro experiments by coculturing 4T1 cells with these two neutrophils, respectively (Supplementary Fig. 8c, d).

Previous studies have confirmed that neutrophil extracellular traps (NETs), which may be induced by bacterial factors, can promote tumor metastasis.^20^ Therefore, we hypothesized that CI group mice with chronic pulmonary *P. aeruginosa* infection would produce more NETs in the lungs than those in the CTRL group. Unsurprisingly, immunofluorescence (IF) staining of lung tissue showed that the CI group expressed higher levels of citH3, which is a hallmark of NETs, than the CTRL group (Supplementary Fig. 9a, b). However, which neutrophil subset releases NETs remains unclear. Thus, we detected the capacity of MHCII^hi^ and MHCII^low^ neutrophils to release NETs. Both neutrophils were isolated by flow sorting and stimulated with or without LPS for 3 h. Immunofluorescence analysis showed upregulated citH3 expression in MHCII^hi^ neutrophils (Fig. 5e). Interestingly, this result was accompanied by the expansion of MHCII^hi^ neutrophils. Consistent with these results, the free dsDNA level released by MHCII^hi^ neutrophils was higher than that released by MHCII^low^ neutrophils (Fig. 5f). These findings indicated that compared to MHCII^low^ neutrophils, MHCII^hi^ neutrophils have both direct and indirect tumor-promoting functions.

Next, we validated whether MHCII^hi^ neutrophils exhibit cancer-promoting functions in vivo. For this purpose, we isolated MHCII^hi^ and MHCII^low^ neutrophils from the lung tissues of CI tumor-bearing mice. Then, neutrophils or PBS were delivered into the lungs while keeping the mouse tongue extended until the liquid was inhaled into the lungs on day 11, 14, and 17 after 4T1 tumor cell inoculation (Fig. 5g). On day 21, the mice were sacrificed, and the lungs were fixed in Bouin’s solution. Then, the metastatic nodules on the lung among the three groups were counted. The results showed that the number of metastatic lung nodules was significantly increased in the MHCII^hi^ group compared to the other groups (Fig. 5g and Supplementary Fig. 10). Moreover, there was no statistically significant difference in the number of metastatic lung nodules between the MHCII^low^ group and the PBS group (Fig. 5h).

### CI-derived CCL2 induces MHCII^hi^ neutrophil accumulation and the formation of a metastatic niche in the lung

**Fig. 6:**
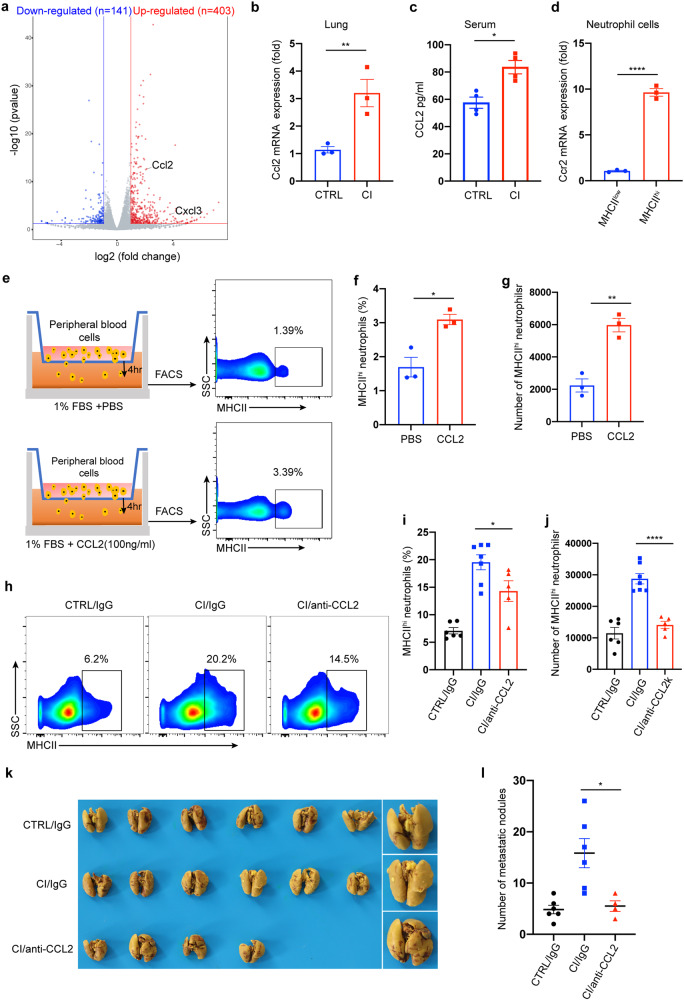
Chronical pulmonary infection-derived CCL2 recruits MHCII^hi^ neutrophils into the lung. **a** Volcano plot showing differential gene expression between lung tissue of tumor-free mice with or without chronic PAO1 infection. Genes with a false discovery rate (FDR) of <5% and an absolute fold change (FC) of >2 are highlighted in blue and red, denoting down- and upregulated genes, respectively, in the lung tissues from the two groups. *n* = 2 mice/group. **b**, **c** 4T1-bearing mice were treated with or without chronic PAO1 infection. Real-time PCR was performed on total RNA extracted from the lungs of mice to analyze Ccl2 gene expression (**b**), and the CCL2 protein levels from the serum of mice were measured by ELISA (**c**). **d** The levels of Ccr2 mRNA in MHCII^hi^ or MHCII^low^ neutrophils in the lung were measured by real-time PCR. **e**–**g** Experimental design of the Transwell migration assay. All blood cells except erythrocytes derived from tumor-bearing mice were added to the top chamber (1 × 10^6^ cells), and the chemoattractant CCL2 (100 ng/ml) or control PBS was added to the bottom chamber (**e**). After incubation for 4 h, the cells harvested from the lower chamber were analyzed using flow cytometry. The proportion (**f**) and number (**g**) of MHCII^hi^ neutrophils. **h**–**j** Representative dot plots showing the exchange in the proportion of MHCII^hi^ neutrophils in the lung tissue of mice after neutralizing CCL2 (**h**). After the establishment of 4T1-bearing mouse models with or without chronic PAO1 infection, neutralizing antibody against CCL2 or isotype control (IgG) at 40 μg per mouse was instilled into the mouse via the trachea. The mice were sacrificed 2 days after the dose (day 18), and the proportion (**i**) and number (**j**) of MHCII^hi^ neutrophils in the lungs of mice were determined by FACS analysis. **k**, **l** Bright-field imaging of metastatic nodules in the lungs of tumor-bearing mice after neutralizing CCL2 (**k**). After the establishment of 4T1-bearing mouse models with or without chronic PAO1 infection, the CCL2-neutralizing antibody and isotype control at 40 μg per mouse were instilled into the lungs of mice on days 11, 14, and 17. On day 21, the mice were sacrificed, and the metastatic nodules in the lung were counted (**i**). *n* = 4–6 mice/group. All data are presented as the mean ± SEM. Statistical values were calculated using one-way ANOVA (**b**, **c**, **d**, **f**, **g**, **l**) or an unpaired *t* test (**i**, **j**). ns not significant; ****P < 0.0001; **P < 0.01; *P < 0.05

To further explore the mechanism of MHCII^hi^ neutrophil accumulation in the lung, we analyzed the transcriptomic data from lung tissues of uninfected and chronically bacteria-infected mice (Fig. 6a). Chemokines and cytokines are important mediators that regulate the migration and infiltration of immune cells. A number of chemokines were highly upregulated in chronically infected mice (Supplementary Fig. 11a). Therefore, we focused on the upregulated chemokines. CCL2 has long been known to recruit monocytes/macrophages.^21^ Interestingly, CCL2 can also recruit neutrophils through CCL2-CCR2 signaling in an inflammatory state.^22^ These previous studies prompted us to investigate whether CCL2 is involved in the recruitment of MHCII^hi^ neutrophils to promote breast cancer lung metastasis. We then analyzed the TISIDB database^23^ to determine the functional correlation of Ccl2 with neutrophil abundance in breast cancer patients. We found that Ccl2 expression positively correlated with the number of neutrophils (Supplementary Fig. 11b). Moreover, a strong association between high Ccl2 expression and poor survival in patients with breast cancer (triple negative) was observed when we used a larger patient dataset from KMPlotter^24^ (*n* = 126; Supplementary Fig. 11c). Increased RNA and protein expression levels of CCL2 were detected in the lung tissues and peripheral blood of chronically infected mice, respectively (Fig. 6b, c). Moreover, compared to MHCII^low^ neutrophils, MHCII^hi^ neutrophils expressed higher levels of Ccr2 (Fig. 6d).

Subsequently, we assessed the ability of CCL2 to recruit MHCII^hi^ neutrophils in vitro. Peripheral blood cells derived from chronically bacteria-infected mice after red blood cell lysis were added to the Transwell upper chamber in serum-free medium, and the lower chamber was filled with medium containing 1% FBS with or without CCL2. After resting in culture for 4 h, the cells in the bottom chamber were collected for FACS analysis (Fig. 6e). The results revealed that the proportion of MHCII^hi^ neutrophils in the total neutrophil fraction was significantly increased after CCL2 induction (Fig. 6f), and the absolute numbers of MHCII^hi^ neutrophils were also increased remarkably after chemokine induction (Fig. 6g). Furthermore, to exclude interference from other blood cells, we isolated MHCII^hi^ neutrophils from the peripheral blood of CI tumor-bearing mice. Then, we repeated the above experiment by replacing total blood cells with MHCII^hi^ neutrophils. Similarly, more cells migrated to the lower compartment following CCL2 treatment (Supplementary Fig. 11d).

In addition, we asked whether CCL2 recruits MHCII^hi^ neutrophils in vivo. The tumor-bearing mouse models were chronically infected with bacteria. Then, neutralizing antibodies against CCL2 or isotype control (IgG) were instilled into the mouse lung via the trachea. The mice were sacrificed 2 days after the last dose (day 18), and the proportion and number of MHCII^hi^ neutrophils in the lungs of mice were evaluated via FACS analysis (Fig. 6h). The results showed that the CCL2-neutralizing antibody significantly inhibited MHCII^hi^ neutrophil migration toward the lung (Fig. 6i, j). This observation is consistent with our in vitro experiments. It has been reported that CCL2 can recruit monocytes/macrophages to facilitate the metastasis of breast cancer.^25,26^ Here, we attempted to verify whether CCL2 plays an important role in mouse models of breast cancer lung metastasis with chronic infection. The CCL2-neutralizing antibody and isotype control were instilled into the lungs of mice on days 11, 14, and 17 after mouse model establishment. On day 21, the mice were sacrificed, and the metastatic nodules on the lung were counted (Fig. 6k). The results showed that the number of metastatic lung nodules was significantly decreased in the anti-CCL2 group compared to the IgG group (Fig. 6l). Overall, these results suggest that chronic bacterial infection in the lung upregulates CCL2 expression, and in turn, CCL2 can partially recruit MHCII^hi^ neutrophils to the lung to promote lung metastasis of breast cancer.

Interestingly, the transcriptomic data showed that CXCL3 was also highly expressed in chronically bacterial-infected lung tissues. Although a few studies have reported the role of CXCL3 in breast cancer lung metastasis, our study confirmed that the blockade of CXCL3 could suppress the metastasis of breast cancer to the lung in vivo (Supplementary Fig. 12a, b). In addition, CXCL3-neutralizing antibody significantly suppressed the migration of neutrophils to the lung, as previously reported^27,28^ (Supplementary Fig. 12c, d), but did not decrease the proportion of MHCII^hi^ neutrophils (Supplementary Fig. 12e, f). Collectively, these data strongly indicate that CCL2, rather than CXCL3, recruits MHCII^hi^ neutrophils to promote lung metastasis of breast cancer. Thus, CCL2 may be a potential prognostic marker for lung metastasis in breast cancer patients.

## Discussion

The majority of breast cancer deaths occur as a result of distant metastasis,^29–31^ yet the exact mechanisms underlying tumor metastasis are poorly understood. The lung, as a mucosal tissue, is constantly exposed to many airborne microorganisms and easily colonized by diverse pathogenic bacterial communities in immunocompromised conditions, such as patients with cancer.^7,32,33^ Thus, the development of lung metastasis in breast cancer may be influenced by pathogens in the lung indirectly or directly through regulation of the immune response. Although some literatures have reported that bacteria may promote lung metastasis of breast cancer, the effects of bacteria during chronic pulmonary infection on breast cancer lung metastasis remain unclear. Here, our findings demonstrate that chronic inflammation caused by bacteria can enhance breast cancer lung metastasis by recruiting tumor-promoting MHCII^hi^ neutrophils via the chemokine CCL2.

Although there is growing evidence that the microbiota is involved in tumorigenesis and therapeutic response, the current literature largely focuses on the gut microbiota. Whether and how the lung microbiota, such as pathogenic bacteria, influence tumor progression and metastasis has not been clearly elucidated. Our data showed that chronic, but not acute, pulmonary bacterial infection caused by *P. aeruginosa* and *S. aureus* can promote breast cancer lung metastasis in mice. Unlike acute infection, chronic bacterial infection in the lung did not recruit a large number of neutrophils but did recruit abundant MHCII^hi^ neutrophils. This result suggests that MHCII^hi^ neutrophils are potentially involved in the changes in the lung immune microenvironment induced by chronic infection. The tumor-promoting phenotype of MHCII^hi^ neutrophils along with previous research shows that neutrophils can augment breast cancer lung metastasis and colonization.^34–37^ Our results showed that MHCII^hi^ neutrophils exhibited more robust tumor-promoting effects than their MHCII^low^ counterparts. Tumor metastasis is a complex process, and our results showed that MHCII^hi^ neutrophils performed protumor functions in the lungs of mice. However, it is not known whether MHCII^hi^ neutrophils affect the other steps of tumor metastasis. This interesting hypothesis requires further experimentation in future research. Overall, these data support the idea that neutrophils encompass phenotypically and functionally distinct subsets in various cancers and may provide a new way to manipulate and investigate the neutrophil response in cancer therapy.

Neutrophils are often known for their role in microbiota resistance, but accumulating evidence suggests that these cells can oppose or potentiate cancer progression in tumor-bearing hosts. One possible reason for the contradictory function is the existence of phenotypic heterogeneity and functional diversity within neutrophils that determine their response to different cancer environments.^9,38,39^ Similar to macrophages, neutrophils are highly capable of polarization plasticity. Previous studies have identified distinct populations of circulating neutrophils (N1 and N2) present in the peripheral blood.^37,38^ TGF-β can drive neutrophils to convert neutrophils to the N2 phenotype (a protumor phenotype similar to M2 macrophages),^40^ and chronic exposure to nicotine also skews neutrophils toward the N2 phenotype.^2^ Our data showed that N2 neutrophils dramatically accumulated in the lungs of tumor-bearing mice after chronic PAO1 infection. Although N2 neutrophils are a small proportion of the total neutrophils present after infection (approximately 2%), this study suggests that chronic inflammation caused by PAO1 infection can immune-train neutrophils to the tumor-promoting phenotype N2. Moreover, a growing body of literature focuses on the heterogeneity of neutrophils in various cancer types, such as SiglecF^hi^ neutrophils in lung adenocarcinoma,^41^ CD62L^dim^ neutrophils in breast cancer,^42^ CD16^high^ CD62L^dim^ neutrophils in head and neck squamous cell carcinoma (HNSCC),^43^ and CD66^+^ neutrophils in hepatocellular carcinoma (HCC).^44^ Different phenotypes of neutrophil subsets may have varying functions at different stages of tumorigenesis and development, from tumor initiation to primary tumor growth to metastasis, in diverse kinds of tumor types. Our CyTOF results revealed that there were defined MHCII^hi^ neutrophils present in the lungs of tumor-bearing mice, which accounted for approximately 5% of total neutrophils and approximately 3% of neutrophils in the peripheral blood of tumor-bearing mice. After chronic *P. aeruginosa* or *S. aureus* infection, the proportion of these cells increased to approximately 15%. These results strongly suggest that MHCII^hi^ neutrophils play an important role in the initiation and progression of breast cancer lung metastasis. Collectively, these findings led us to question whether chronic inflammation caused by other endogenous or exogenous factors can also recruit MHCII^hi^ neutrophils into the lung to support tumor development.

Spontaneous tumor regression was observed in some patients with severe bacterial infection as early as the 19^th^ century. Thus, many attempts have been made to utilize bacteria for tumor therapy,^45–48^ and some beneficial results have been achieved. Our results (Supplementary Fig. 1e, f) are consistent with the finding that transient acute bacterial infection can inhibit tumor metastasis. However, another major issue is the fact that ~20% of all human malignancies are related to infection with bacteria, viruses and parasites.^49^ It is noteworthy that most bacteria associated with the development of cancer often persist for a long time in the host, which constantly contributes to chronic inflammation. Bacteria can manipulate tumor progression directly or indirectly by regulating the host immune response and remodeling the tumor microenvironment or premetastatic niches. For instance, long-term persistence of bacteria in the lung can accelerate the development of lung cancer via γδ T cells.^7^ This study identified that chronic *P. aeruginosa* (representative gram-negative bacteria) or *S. aureus* (typical Gram-positive bacteria) infection in the lung facilitates breast cancer lung metastasis. Therefore, it is highly likely that other chronic pathogenic bacterial infections also promote breast cancer lung metastasis. Furthermore, the virulence factors associated with chronic infection have not been investigated. These remaining interesting questions need to be addressed in future studies.

Multiple chemokines can recruit neutrophils into specific tissues. Among these cytokines, CXCL3 has been reported to be involved in the chemotaxis of neutrophils.^28^ Our results again confirmed this conclusion. Although CXCL3 did not specifically recruit MHCII^hi^ neutrophils to the lungs, blocking CXCL3 to suppress lung metastasis of breast cancer is an interesting and valuable topic worthy of further verification. Importantly, we found that CCL2 can recruit MHCII^hi^ neutrophils into the lung. CCL2, classified as a Ccr2 ligand, has been reported to play a key role in the chemotaxis and activation of monocytes and macrophages, while little attention has been given to neutrophils.^50,51^ One potential reason is that MHCII^hi^ neutrophils account for only a relatively small population of the total neutrophils, especially in the healthy state. However, CCL2 may recruit MHCII^hi^ neutrophils through other mechanisms independent of CCR2. Therefore, a better understanding of how CCL2 recruits MHCII^hi^ neutrophils requires more research. In addition to the effects of CCL2 and CCR2, it is possible that additional components (e.g., ligand‒receptor pairs) driven by chronic inflammation can also recruit MHCII^hi^ neutrophils, which remain to be identified.

**Fig. 7:**
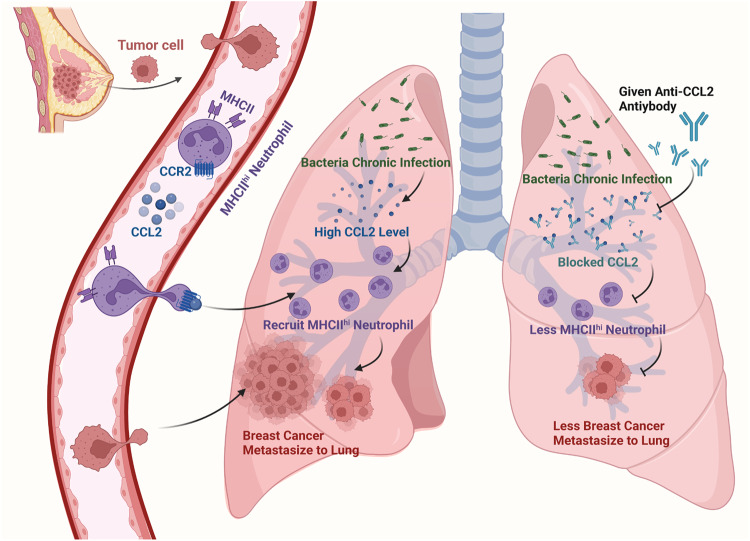
Schematic representation of the proposed mechanism of chronic pulmonary bacterial infection-induced lung metastasis. Schematic was created with BioRender (www.biorender.com)

In summary, our findings reveal that chronic bacterial infection can promote breast cancer lung metastasis by recruiting MHCII^hi^ neutrophils into the premetastatic lung via the chemokine CCL2 (Fig. 7). Specifically, considering immune cells as critical therapeutic targets, our study posits MHCII^hi^ neutrophils as promising candidates for cancer therapy. Given the convenience of diagnosis, CCL2 may be a promising clinical diagnostic marker for lung metastasis of breast cancer.

## Materials and methods

### Bacterial strains and mammalian cell lines

*Pseudomonas aeruginosa* strain PAO1 was provided by S. Lory (Harvard Medical School), and *Staphylococcus aureus* subsp. *aureus* strain Seattle 1945 was purchased from American Type Culture Collection (ATCC, 25923). PAO1 and Seattle 1945 were grown overnight in LB (Luria-Bertani) broth with shaking (220 r.p.m.) at 37 °C. The mouse breast carcinoma cell Line 4T1 was obtained from ATCC and transduced with firefly luciferase lentiviral expression particles (HanBio, Shanghai, China) to generate 4T1-Luc cells. 4T1 and 4T1-Luc cells were maintained in RPMI-1640 (Gibco) supplemented with 10% FBS (Gibco), streptomycin (100 μg/ml), and penicillin (100 units/ml).

### Mice and ethics statement

Female FVB/NJGpt-Tg(MMTV-PyMT)/Gpt mice were purchased from GemPharmatech Co., Ltd. Five- to six-week-old female BALB/c mice were purchased from Beijing HFK Bioscience Co. All mice were housed in a specific pathogen-free facility at the State Key Laboratory of Biotherapy, Sichuan University. All mouse experiments were reviewed and approved by the Institutional Animal Care and Use Committee of Sichuan University (2021559A).

### Human samples and ethics statement

Human lung puncture samples of patients with breast cancer (tissue, BAL fluid and plasma) were obtained from the Sichuan Provincial People’s Hospital, University of Electronic Science and Technology of China. Written informed consent was obtained from all patients, and the study was approved by the local ethics committee of the Sichuan Provincial People’s Hospital (SPPH202304).

### Animal experiments

To examine the effect of acute or chronic bacterial infection, we constructed a spontaneous metastasis model. In brief, luciferase-labeled 4T1 tumor cells (2 × 10^5^ cells in 100 μl PBS) or WT 4T1 cells (2 × 10^5^ cells in 100 μl PBS) were injected into the fourth mammary fat pad of 5–6-week-old BALB/c mice. Seven days after implantation, the lungs of mice were acutely or chronically infected with PAO1 or SA. The experimental setup of lung infection was as follows. After the tongues of anesthetized mice were kept extended with forceps, planktonic bacterial cells in 50 µl PBS (acute infection) or agar bead-embedded bacterial cells in 50 µl PBS (prepared as previously described,^13^ chronic infection) were delivered into the oropharyngeal cavity until the solution was completely aspirated into the lungs. For the spontaneous breast cancer MMTV-PyMT mouse model, some mice were chronically pulmonary infected with PAO1 beads as a CI group at 8 weeks and 12 weeks of age, and the other mice served as the CTRL group. At the endpoint, mice were sacrificed, the numbers of metastatic nodules on the lungs were counted, or the presence of metastatic lesions was evaluated using ex vivo bioluminescent imaging and histological analysis. Images were analyzed using a Xenogen IVIS imaging system (Caliper Life Science). For experiments involving the functional validation of MHCII^hi/low^ neutrophils in vivo, MHCII^hi/low^ neutrophils (3 × 10^5^ cells in 50 μl PBS) isolated from the lungs of tumor-bearing mice were delivered into the trachea of 4T1 tumor-bearing mice after implantation for 1 week on days 11, 14, and 17 by the method described above. At the endpoint, the mice were sacrificed, and the lung metastatic nodules were counted. To explore the effect of CCL2 and CXCL3 on the recruitment of MHCII^hi^ neutrophils and breast cancer lung metastasis, mice bearing 4T1 tumors were randomly divided into three groups one week after implantation. The two groups were both infected with PAO1 beads (chronic infection) and subsequently administered IgG or CCL2/CXCL3-neutralizing antibodies through the trachea at predetermined time points. The third group was administered PBS and IgG. At the endpoints, the mice were sacrificed, and the lungs were collected for nodule count or FACS analysis.

### Chemotaxis assay

A chemotaxis assay was performed to assess the chemotaxis of neutrophils induced by CCL2. White blood cells in the peripheral blood or MHCII^hi^ neutrophils isolated from the lungs of mice were seeded into the upper chamber in 100 μl of serum-free RPMI-1640 medium, and the chemoattractant CCL2 (100 ng/ml) or control PBS was added to the bottom chamber in RPMI-1640 medium containing 10% FBS. After incubation for 4 h, the cells harvested from the lower chamber were analyzed by flow cytometry or counted using a hemocytometer.

### Flow cytometry and FACS sorting

Single-cell suspensions were obtained from lung tissue and peripheral blood. Briefly, the lungs of tumor-bearing or tumor-free mice with or without pulmonary chronic PAO1 infection were placed in a gentle MACS™ C tube (Miltenyi Biotec) and mechanically homogenized in 4 ml RPMI-1640 and 10% FBS containing 0.2 mg/ml collagenase type I/IV using a gentle MACS™ Octo Dissociator with Heaters following the manufacturer’s protocol (Miltenyi Biotec). Digested lung tissues were gently meshed through 70 μM cell strainers using a plunger. Red blood cells were removed using 2 ml red blood cell lysis buffer (Solarbio, catalog no. R1010) per cell pellet for 5 min (for lung cells) or 15 min (for peripheral blood). The resulting single-cell suspensions were washed with PBS and resuspended in PBS containing 1% FBS. For cell surface marker staining, cell suspensions were incubated with FcR Blocking Reagent (Biolegend, catalog no. 101319) for 15 min at 4 °C, stained with fluorescent conjugated Abs for 30 min at 4 °C and washed twice with PBS. For intranuclear staining, cells were fixed and permeabilized using the eBioscience™ Foxp3/Transcription Factor Staining Buffer Set (eBioscience, catalog no. 00-5523-00). Subsequently, the cells were stained with fluorescent conjugated Abs specific for protein in the nucleus and incubated in the dark for 30 min at 4 °C, followed by washing twice with PBS. Positive staining with the BD fixable viability dye FVS620 (BD Biosciences, catalog no. 564996) was used to exclude dead cells. Data were collected on a BD FACSymphony analyzer (BD Biosciences) and analyzed with FlowJo software (v.10.4 for Mac OS X). Cell sorting was performed with a FACSAria III instrument (BD Biosciences). The following Abs were all purchased from Biolegend: CD45 (clone S18009, catalog no. 157214); CD3 (clone 17A2, catalog no. 100205); PD-1 (clone 29 F.1A12, catalog no. 135210); F4/80 (clone BM8, catalog no. 123109); CD11b (clone M1/70, catalog no. 101212); CD206 (clone C068C2, catalog no. 141716); CD86 (clone GL-1, catalog no. 105013); Ly6G (clone 1A8, catalog no. 127616); MHCII (clone M5/114.15.2, catalog no. 107608); and Ki67 (clone 11F6, catalog no. 151210).

### ELISA and RT‒qPCR

CCL2 (MCP-1) protein levels in the serum of tumor-bearing mice with or without pulmonary chronic PAO1 infection were measured using a mouse MCP-1 ELISA kit (NeoBioscience, catalog no. EMC113.96) according to the manufacturer’s instructions. Total RNA was isolated from lung tissues or FACS-purified cells using TRIzol reagent (Invitrogen, catalog no. 15596026). cDNA was synthesized from RNA with the PrimeScript^TM^ RT Reagent Kit with gDNA Eraser (Takara, catalog no. RR047A). For real-time qPCR analysis, Ccl2 and Ccr2 expression was analyzed in triplicate and normalized to GAPDH expression with TB Green^®^ Fast qPCR Mix (Takara, catalog no. RR430A) on a LightCycler 96 Real-time PCR System (Roche).

### Histology and immunohistochemistry (IHC)

Lung lobes of tumor-bearing mice subjected to various treatments were fixed in 4% paraformaldehyde overnight and embedded in paraffin following standard procedures. Tissue sections (4 μM) were dewaxed and rehydrated. For histological analysis of tumor burden in mice, lung tissue sections were stained with hematoxylin and eosin (H&E) to define tumor tissue areas in the lung. For immunohistochemical analysis of specific antigens in the lung, the tissue sections were heated at 120 °C for 5 min using a pressure cooker in 10 mM sodium citrate buffer (pH 6.0) for antigen retrieval. The sections were then treated with 3% H_2_O_2_ solution in PBS at room temperature for 10 min to block endogenous peroxidase activity and further blocked with 5% normal goat serum for 1 h at room temperature. The sections were incubated with anti-MHCII antibodies (Invitrogen, catalog no. 14-5321-82) overnight at 4 °C. The tissue sections were subsequently washed with PBS and incubated with biotin-conjugated secondary antibodies for 30 min and then with horseradish peroxidase streptavidin (HRP streptavidin) for 30 min (SPlink Detection Kits; ZSGB-BIO, SP-9001). The sections were developed using the 3,3ʹ-diaminobenzidine (DAB) substrate kit (ZSGB-BIO, ZLI-9017) and counterstained with hematoxylin. Images were captured using an Olympus BX600 microscope and SPOT Flex camera.

### Immunofluorescence (IF) and confocal imaging

Paraffin-embedded sections obtained from the mouse metastatic lung or human metastatic lung were subjected to immunofluorescence (IF) staining according to a standard immunofluorescence paraffin-embedded tissue staining protocol. In brief, after lung tissue sections were dewaxed in xylene and rehydrated, antigen retrieval was carried out in 10 mM sodium citrate (pH 6.0) at 120 °C for 5 min using a pressure cooker. The tissue sections were then blocked with 5% normal goat serum for 1 h at room temperature and further incubated with primary antibodies diluted in blocking buffer overnight at 4 °C. The following antibodies were used for staining: Ly6G (Biolegend, catalog no. 127602), MPO (R&D Systems, catalog no. AF3667), and MHCII (Invitrogen, catalog no. 14-5321-82). The tissue sections were subsequently washed with PBS and incubated with fluorophore-labeled, appropriate secondary antibodies for 1 h at room temperature. The secondary fluorescent antibodies used included donkey anti-goat (Proteintech, catalog no. SA00003-3) and donkey anti-rat (Proteintech, catalog no. SA00007-7) antibodies. DAPI (Vector Laboratories, catalog no. H-2000) was used to mark the nuclei, and fluorescence images were captured by a Nikon confocal A1 laser microscope (Nikon). All image quantification was performed using ImageJ software (version 1.8.0).

### Visualization and quantification of NETs in vitro and in vivo

To examine the capacity of the different neutrophil populations (MHCII^hi^, MHCII^low^ cells) to produce NETs, both neutrophil groups were sorted from lung tissue of tumor-bearing mice by flow cytometry and seeded onto poly-l-lysine-coated slides, which were placed in 24-well plates in advance (2 × 10^5^ cells in 500 μl per well). After centrifugation at 500×*g* for 10 min, *Pseudomonas aeruginosa*-derived LPS was added to the culture medium at a final concentration of 90 μg/ml. Cells were statically incubated at 37 °C for 3 h. The supernatant (culture medium) was harvested to detect the neutrophil-free double-stranded DNA level by an EZQuant dsDNA Quantitation Kit (Fluorometric, Biovision, catalog no. K900-2000) according to the manufacturer’s instructions. Cells were then used for immunofluorescence staining to detect NETs. In brief, the cells in a 24-well plate were washed twice with PBS. Then, the cells were fixed with 4% paraformaldehyde for 15 min and permeabilized with 0.5% Triton X-100 for 5 min. Subsequently, the cells were blocked with 10% goat serum at room temperature for 30 min and stained with DAPI, anti-MPO (R&D Systems, catalog no. AF3667), and anti-H3cit (Novusbio, catalog no. NB100-57135) overnight at 4 °C. Then, fluorescent-coupled secondary antibodies, including donkey anti-goat (Proteintech, catalog no. SA00003-3) and donkey anti-rabbit (Proteintech, catalog no. SA00013-8), were used. For lung tissue staining (from the tumor-bearing mice with or without chronic PAO1 infection), the samples were processed as described in the “immunofluorescence method” section, and the antibodies used were the same as described above. The fluorescence images were captured by a Nikon confocal A1 laser microscope (Nikon), and all image quantification was performed by ImageJ software (version 1.8.0).

### Ex vivo ROS activity assay

Neutrophils were analyzed ex vivo for their reactive oxygen species (ROS) level. MHCII^hi^ or MHCII^low^ neutrophils derived from the lungs of tumor-bearing mice were sorted as described in the flow cytometry methods section. Then, the cells were washed and resuspended in serum-free RPMI-1640 medium, and 10 μM DCFH-DA (Beyotime Biotechnology, catalog no. S0033S) was added for 20 min at 37 °C. Cells were washed with PBS three times to remove DCFH-DA that did not enter the cells. Then, the cells were resuspended in PBS, and the activated DCF signal was analyzed in the FITC channel on a BD FACSymphony analyzer (BD Biosciences). Rosup was added to cells and served as a positive control.

### Transwell assays

For the Transwell migration and invasion assays, 100 μl of serum-free RPMI-1640 medium containing 1 × 10^5^ 4T1 cells was seeded into the upper chamber without (Transwell migration assay) or with (Transwell invasion assay) Matrigel (Corning, catalog no. 354234), and neutrophils (MHCII^hi^, MHCII^low^, 4 × 10^5^ cells in 500 μl RPMI-1640 medium containing 1% FBS) isolated from the lungs of tumor-bearing mice with pulmonary chronic PAO1 infection were added to the bottom chamber. Following 24 h (for migration assays) or 48 h (for invasion assays) incubation at 37 °C in a 5% CO_2_ incubator, the Transwell membranes were fixed with 4% methanol and stained with 1% crystal violet. Then, 4–5 fields were selected and photographed randomly using an inverted microscope (Nikon). The experiments were performed in triplicate.

### Analysis of public databases

Using the TISIDB database, we performed a correlation analysis between the abundance of neutrophils and Ccl2 mRNA expression in breast invasive carcinoma (BRCA) patients.^23^ For survival analyses, overall survival (OS), stratified by Ccl2 mRNA expression, was presented as Kaplan–Meier plots and tested for significance using log-rank tests. The analysis was performed according to previously described methods.^52^

### Cytometry by time-of-flight (CyTOF)

Lungs collected from tumor-bearing mice with or without pulmonary chronic PAO1 infection were analyzed by time-of-flight mass cytometry. Briefly, lung tissues from tumor-bearing mice with or without pulmonary chronic PAO1 infection were cut into small pieces with scissors and digested (RPMI-1640 containing 0.5 μg/ml collagenase I/IV, 250 μg/mL hyaluronase, 20 μg/mL DNase I) for 1 h at 37 °C while shaking. We pooled the lungs of five mice into one sample. Then, the single-cell suspensions were stained with a panel of 41 antibodies (Supplementary Table 1), and mass-tagged cell barcoding (MCB) was used to label individual cell samples as previously described. Palladium-based mass tag cell barcoding was performed with a double-filtering scheme and single-cell deconvolution algorithm.^53^ Barcoded samples were analyzed on a Helios Mass Cytometer (Fluidigm). The generated files were debarcoded using a doublet filtering scheme with mass-tagged barcodes and then manually gated to retain live, singlet, valid immune cells. To obtain accurate immune subset information, we ran the FLOWSOM or X-shift [Phenograph] algorithm on all samples.

### RNA-seq and expression analysis

Total RNA was extracted from two or three replicate samples. RNA sequencing and data analysis were conducted by Novogene Corporation (Beijing, China). Genes with an adjusted *P* value < 0.05 found by DESeq2 were considered differentially expressed.

### Statistical analysis

All statistical analyses were performed using GraphPad Prism (version 7.0). Data are expressed as the mean ± SEM. The *P* value was calculated by an unpaired Student’s *t* test or ANOVA with corresponding numbers (*n*) as indicated in the figures and corresponding legends. The Kaplan–Meier survival analysis was calculated by the log-rank (Mantel–Cox) test. Significant differences between each group are represented as *P < 0.05, **P < 0.01, ***P < 0.001, ****P < 0.0001 unless otherwise indicated.

### Supplementary information

Supplementary information

## Data Availability

Raw and processed RNA sequencing datasets have been deposited in the NCBI GEO database under accession numbers GSE 218630, GSE 218646 and GSE 192890. Other data supporting the findings can be requested from the corresponding author.
