# Supplementary material for: Chronic pulmonary bacterial infection facilitates breast cancer lung metastasis by recruiting tumor-promoting MHCIIhi neutrophils
Source: Signal Transduct Target Ther. 2023 Aug 11;8:296. doi: 10.1038/s41392-023-01542-0 (PMC10415306; doi:10.1038/s41392-023-01542-0)
Supplement: Supplementary file 1 — Supplementary information [file 41392_2023_1542_MOESM1_ESM.docx]

**Chronic pulmonary** **bacterial infection facilitates breast cancer lung metastasis by recruiting tumor-promoting MHCII^hi^ neutrophils**

Teng Ma^1,5^, Yu Tang^1,5^, Taolin Wang^1^, Yang Yang^1^, Yige Zhang^1^, Ruihuan Wang^1^, Yongxin Zhang^1^, Yi Li^3^, Mingbo Wu^1^, Miao Tang^1^, Xueli Hu^1^, Chaoyu Zou^1^, Yuan Ren^1,2^, Huan Liu^1^, Qianhua Zhang^1^, Heyue Li^1^, Min Wu^4✉^, Jing Li^2✉^, Xikun Zhou^1✉^

^✉^e-mail: minwoo2022@126.com (M.W.), lijing1984@scu.edu.cn (J.L.), xikunzhou@scu.edu.cn (X.Z.)

**This file includes:**

- **Figures. S1 to S12**
- **Tables S1, S2**

**Supplementary Figures and Figure Legends**

**
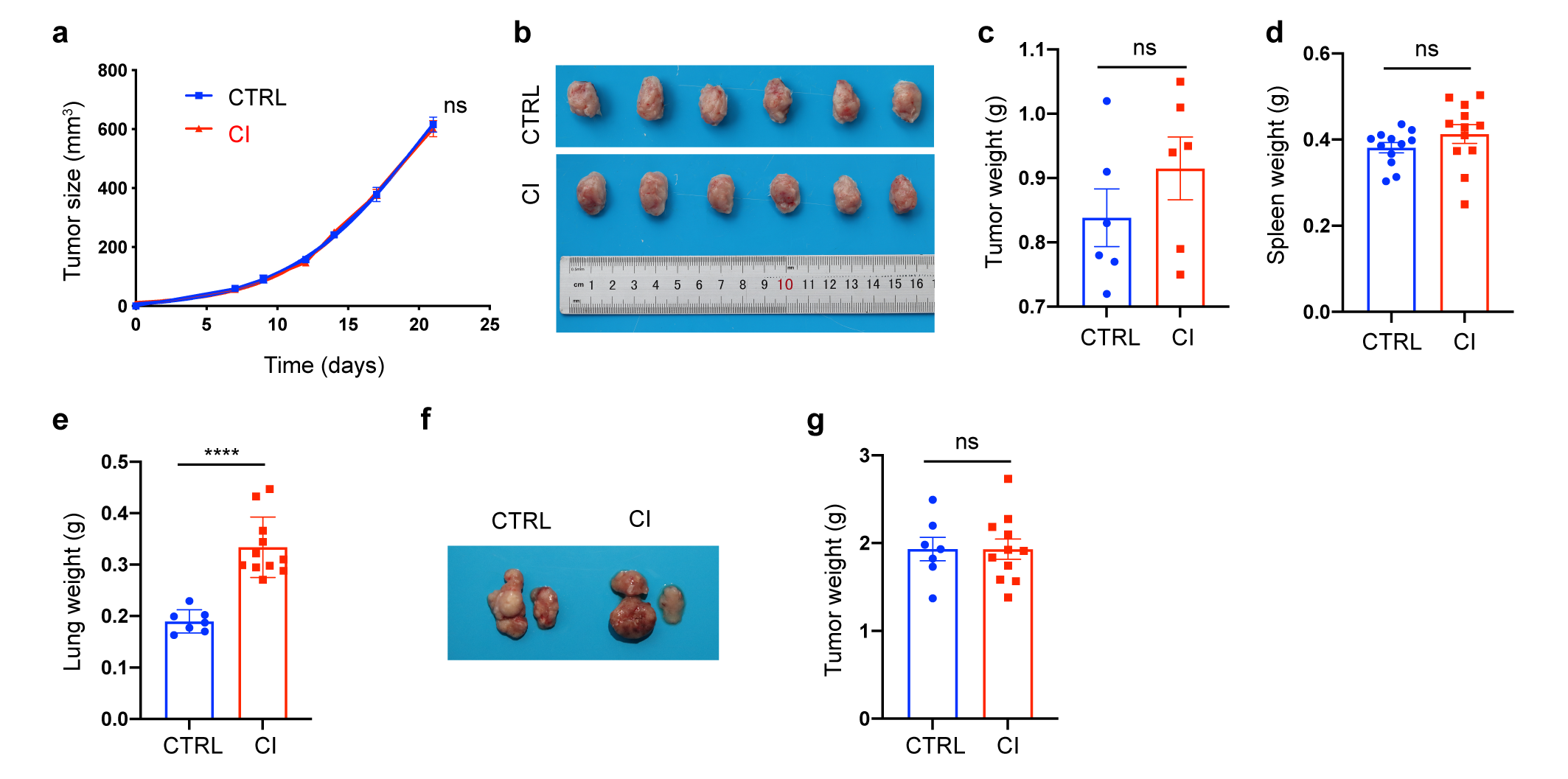
**

**Fig. S1 | Chronic pulmonary infection did not change the development process of the primary tumor. a.** Primary tumor volume of mice bearing 4T1 cells measured after chronic PAO1 infection (n = 10 mice/group). **b-c.** Representative image (**b**) and weight (**c**) of mice bearing tumors after PBS or PAO1-Beads infection at the endpoint (21 days), n = 6 mice/group. **d.** Spleen weight of tumor-bearing mice from the CTRL group (n = 12 mice/group) and CI group (n = 13 mice/group). **e.** Lung weight of 14-week-old MMTV-PyMT mice from the CTRL group (n = 7) and CI group (n = 11). **f-g.** Representative image (**f**) and weight (**g**) of tumors of 14-week-old MMTV-PyMT mice from the CTRL group (n = 7) and CI group (n = 11). All data are presented as the mean ± S.E.M. ﻿Statistical significance was calculated using an unpaired t test. ns, not significant.


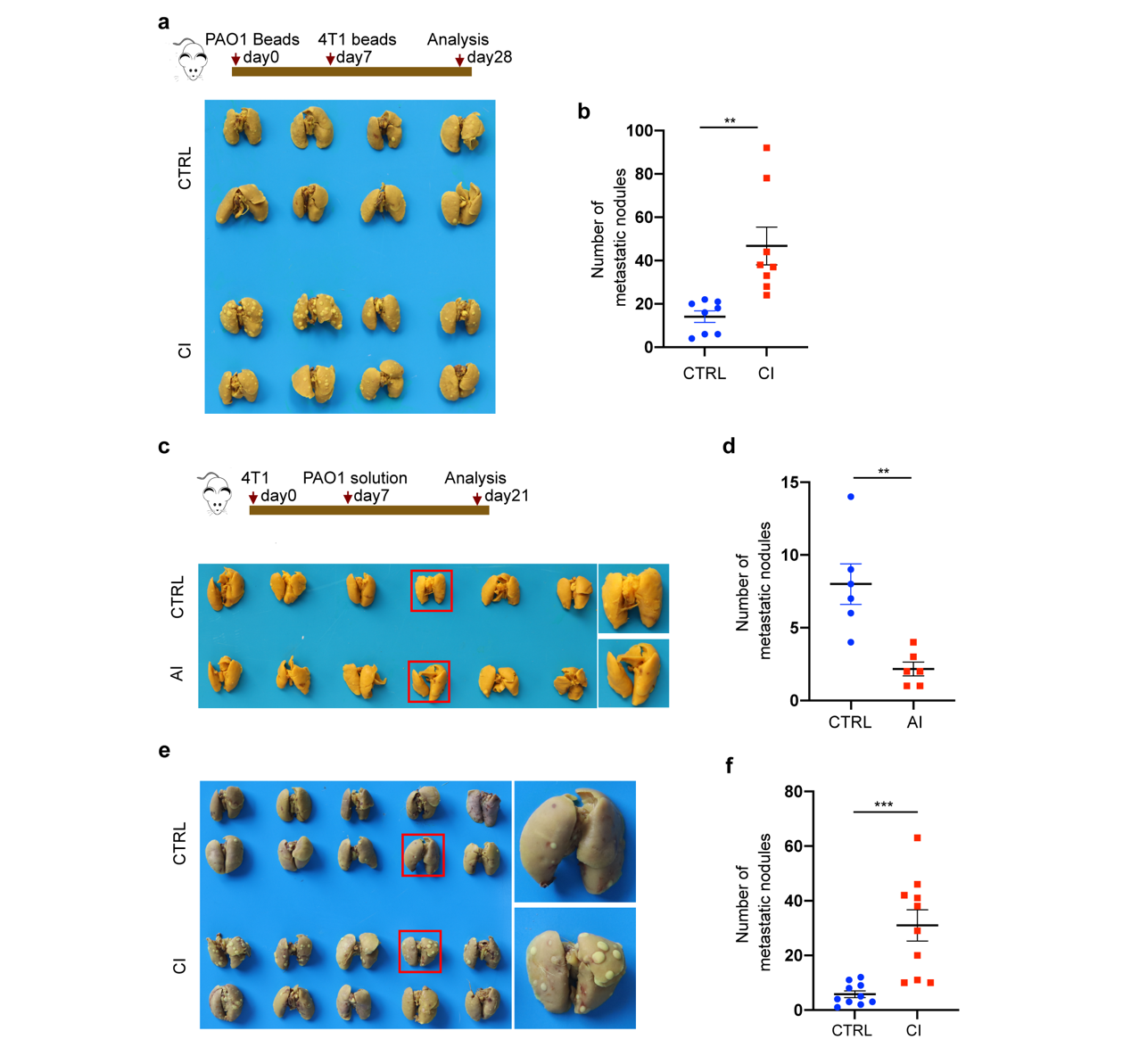


**Fig. S2 | Chronic pulmonary bacterial infection accelerated lung metastasis.** **a-b.** Representative image (**a**) and number of metastatic nodules (**b**) in the lungs. One week after chronic pulmonary PAO1 infection or PBS addition, the two groups of mice were orthotopically inoculated with 4T1 cells. On Day 21 after implantation, the mice were sacrificed, and the lung nodules were counted, n-8 mice/group. **c-d**. Representative image (**c**) and number of metastatic nodules (**d**) in the lungs after PBS or acute PAO1 solution infection (AI), n = 6 mice/group. **e-f.** Image (**e**) and number of metastatic nodules (**f**) in the lungs after PBS or SA-Beads infection, n = 10 mice/group. All data are presented as the mean ± S.E.M. ﻿Statistical significance was calculated using an unpaired t test. ****P* < 0.001; ***P* < 0.01.


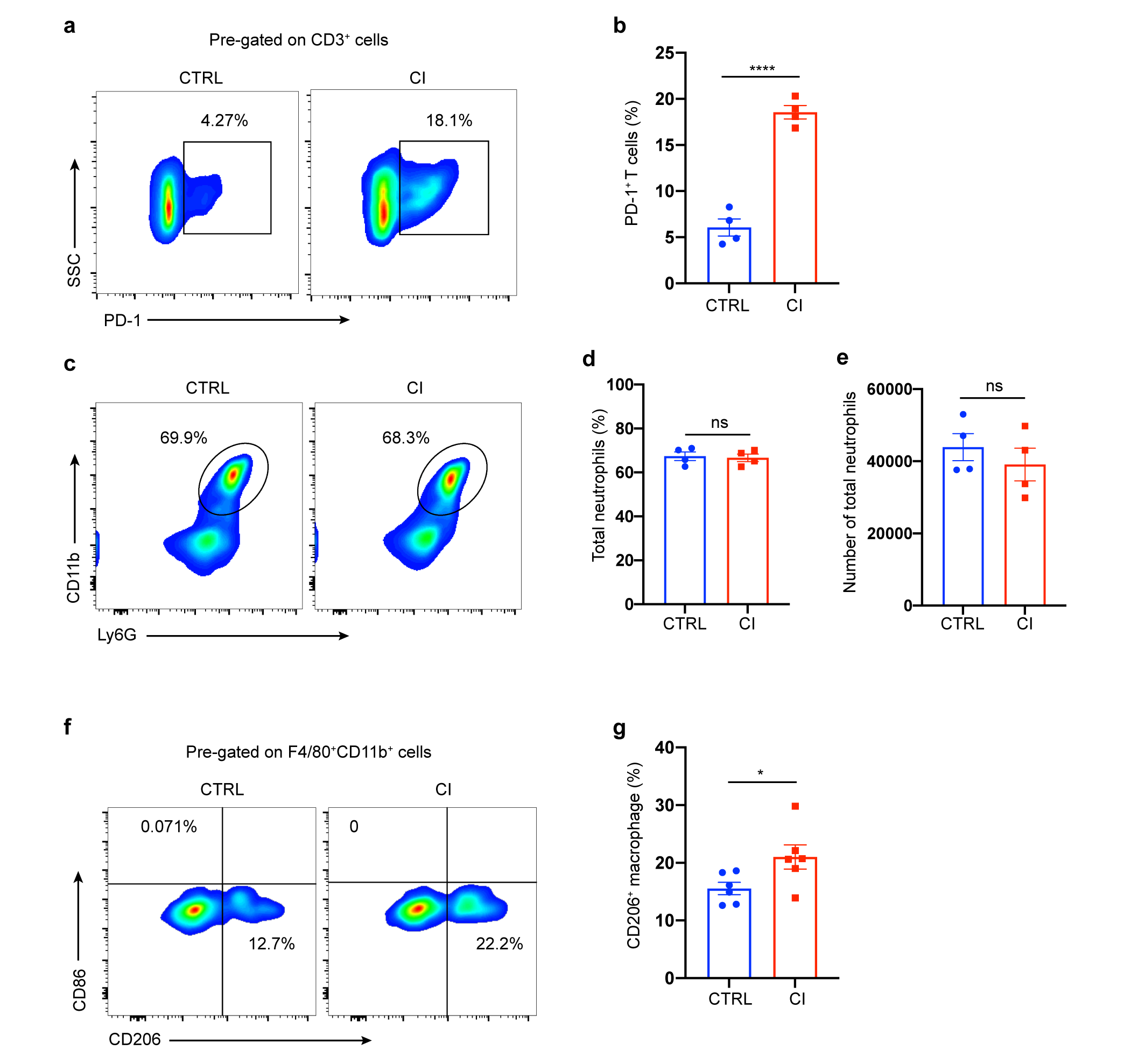


**Fig. S3 | Verification of the changed status of immune cells following chronic pulmonary infection via flow cytometry. a.** Representative flow cytometric dot plots showing CD11b^+^Ly6G^+^ neutrophils derived from the lungs of CTRL and CI group mice. n = 4 mice/group. **b-c.** Quantification of neutrophil numbers (**b**) and frequency (**c**) as in (**a**). n = 4 mice/group. **d-e.** Representative plots (**d**) and frequency (**e**) of PD1^+^CD3^+^ T cells gated on live CD45^+^ cells from the CTRL and CI groups. n = 4 mice/group. **f.** Representative plots showing M1 (CD86) and M2 (CD206) macrophages from the lungs of the two groups. n = 4 mice/group. **g.** Flow cytometry–based quantification of the percentage as in (**f**). n = 4 mice/group. All data are presented as the mean ± S.E.M. ﻿Statistical significance was calculated using an unpaired t test. ns, not significant; ****P < 0.0001; *P < 0.05


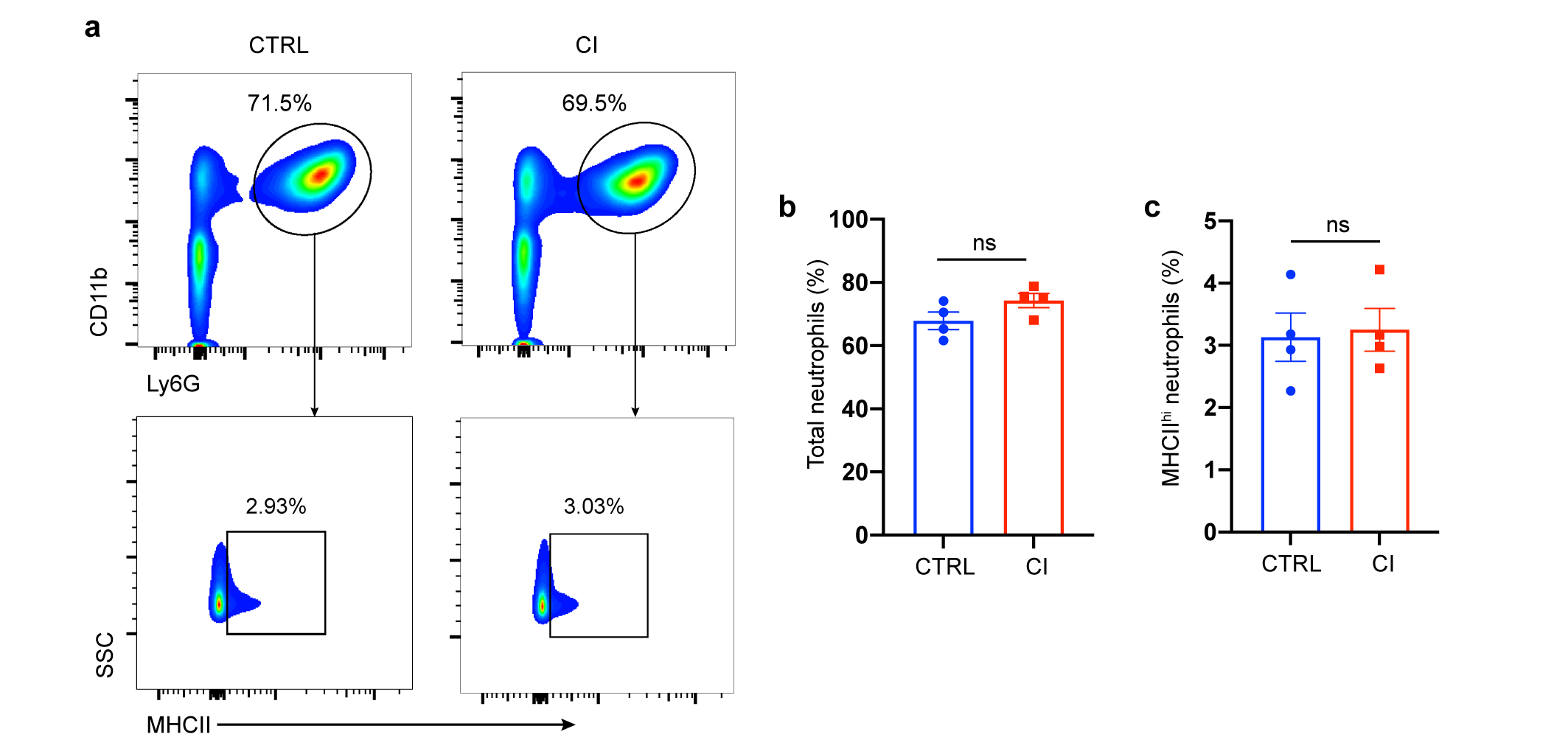


**Fig. S4 | The proportion of MHCII^hi^ neutrophils in the peripheral blood of tumor-bearing mice with or without chronic pulmonary *P. aeruginosa* infection. a.** Representative dot plots showing CD11b^+^Ly6G^+^ neutrophils in live CD45+ cells and MHCII^+^ neutrophils in total neutrophils from the blood of tumor-bearing mice treated with or without PAO1 beads. n = 4 mice/group. **b-c.** Representative flow cytometric analysis (**b**) and quantification (**c**) of total neutrophils and MHCII^hi^ neutrophils derived from Panel **a**. n = 4 mice/group. All data are presented as the mean ± S.E.M. ﻿Statistical significance was calculated using an unpaired t test. ns, not significant.


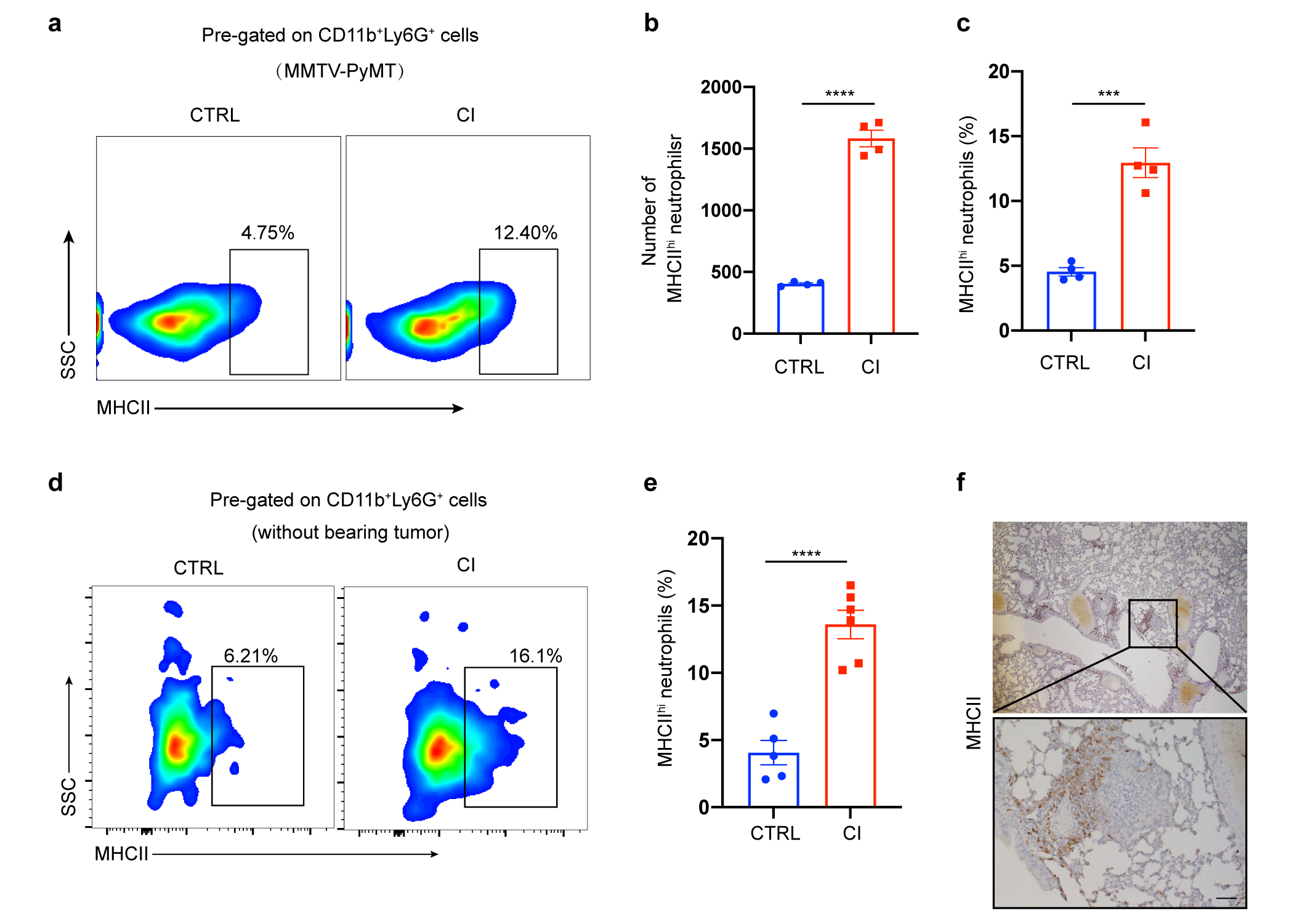


**Fig. S5 | Chronic pulmonary bacterial infection induced MHCII^hi^ neutrophil accumulation in the lung tissues of** **MMTV-PyMT or tumor-free mice.** **a.** Flow cytometry–based detection of MHCII^high^ or MHCII^low^ neutrophils from the lung tissue of MMTV-PyMT mice with or without chronic PAO1 infection. Plots are shown for gated live CD45^+^CD11b^+^Ly6G^+^ cells. **b-c.** Frequency (**b**) and quantification (**c**) of MHCII^hi^ neutrophils in **a.**, n = 4 mice/group. **d-e.** Representative flow cytometric analysis (**d**) and quantification (**e**) of the MHCII^hi^ neutrophil population in total neutrophils derived from the lungs of tumor-free mice with or without chronic PAO1 infection. n = 5 mice/group. **f.** Representative MHCII mAb staining of lung tissue from tumor-bearing mice with chronic PAO1 infection. Scale bar, 50 μm. All data are presented as the mean ± S.E.M. ﻿Statistical significance was calculated using an unpaired t test. *****P* < 0.0001.


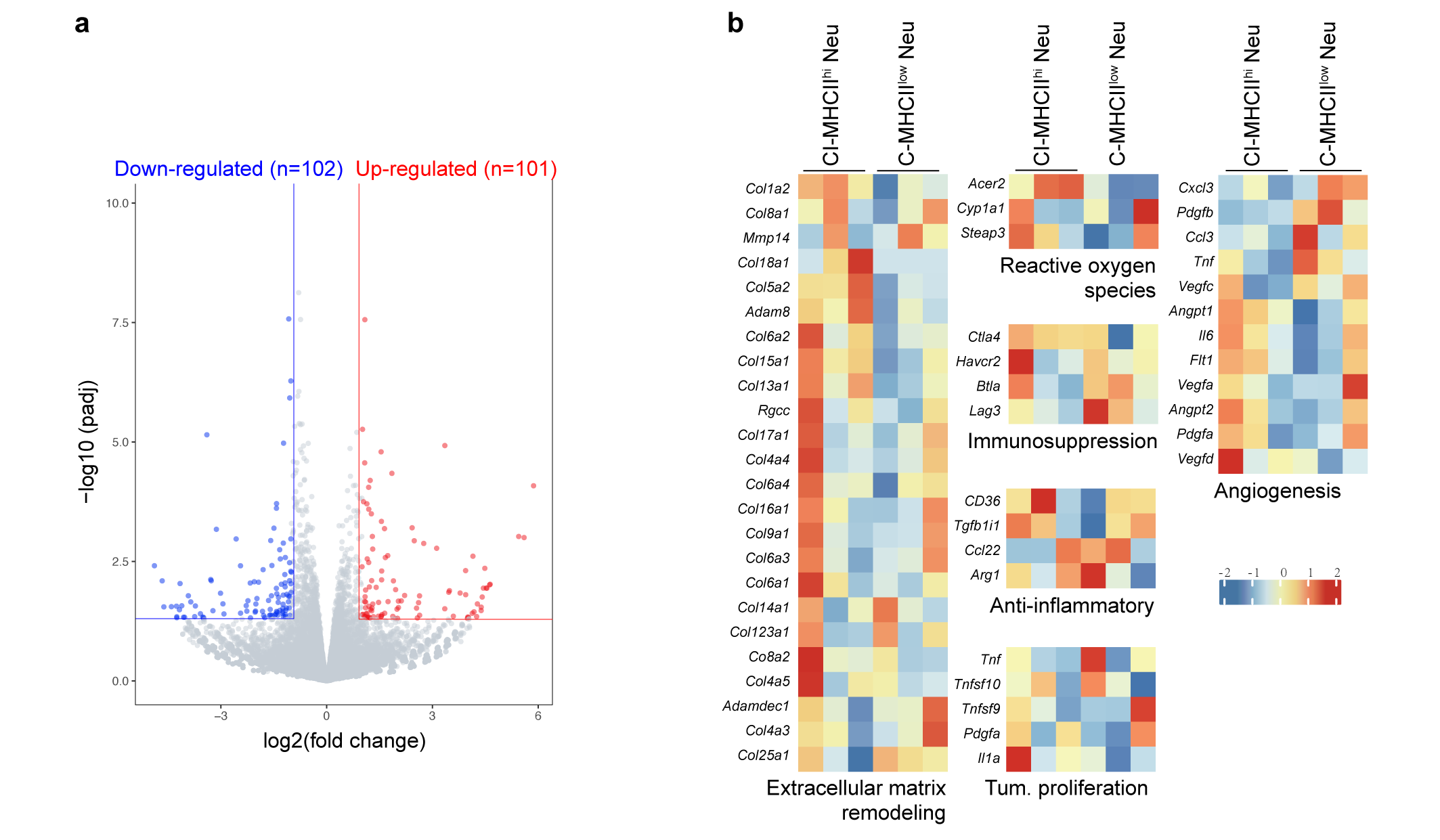


**Fig. S6 | Phenotyping of MHCII^hi^ neutrophils in the lungs from *P. aeruginosa-*infected and uninfected mice by RNA-seq analysis. a.** Volcano plot showing differential gene expression in MHCII^hi^ neutrophils isolated from the lungs of 4T1-bearing mice with or without chronic PAO1 infection. Genes with a fold change higher than 2 and a *P* value of < 0.05 are highlighted in blue and red, denoting down- and upregulated genes, respectively, in the two different sources of neutrophils. **b.** Average expression levels of genes involved in extracellular matrix remodeling, reactive oxygen species, immunosuppression, anti-inflammation, angiogenesis, and tumor proliferation in MHCII^hi^ neutrophils isolated from the lungs of 4T1-bearing mice with or without chronic PAO1 infection. C-MHCII^hi^ Neu: MHCII^hi^ neutrophils from the lungs of 4T1-bearing mice without chronic PAO1 infection. CI-MHCII^hi^ Neu: MHCII^hi^ neutrophils from the lungs of 4T1-bearing mice with chronic PAO1 infection.


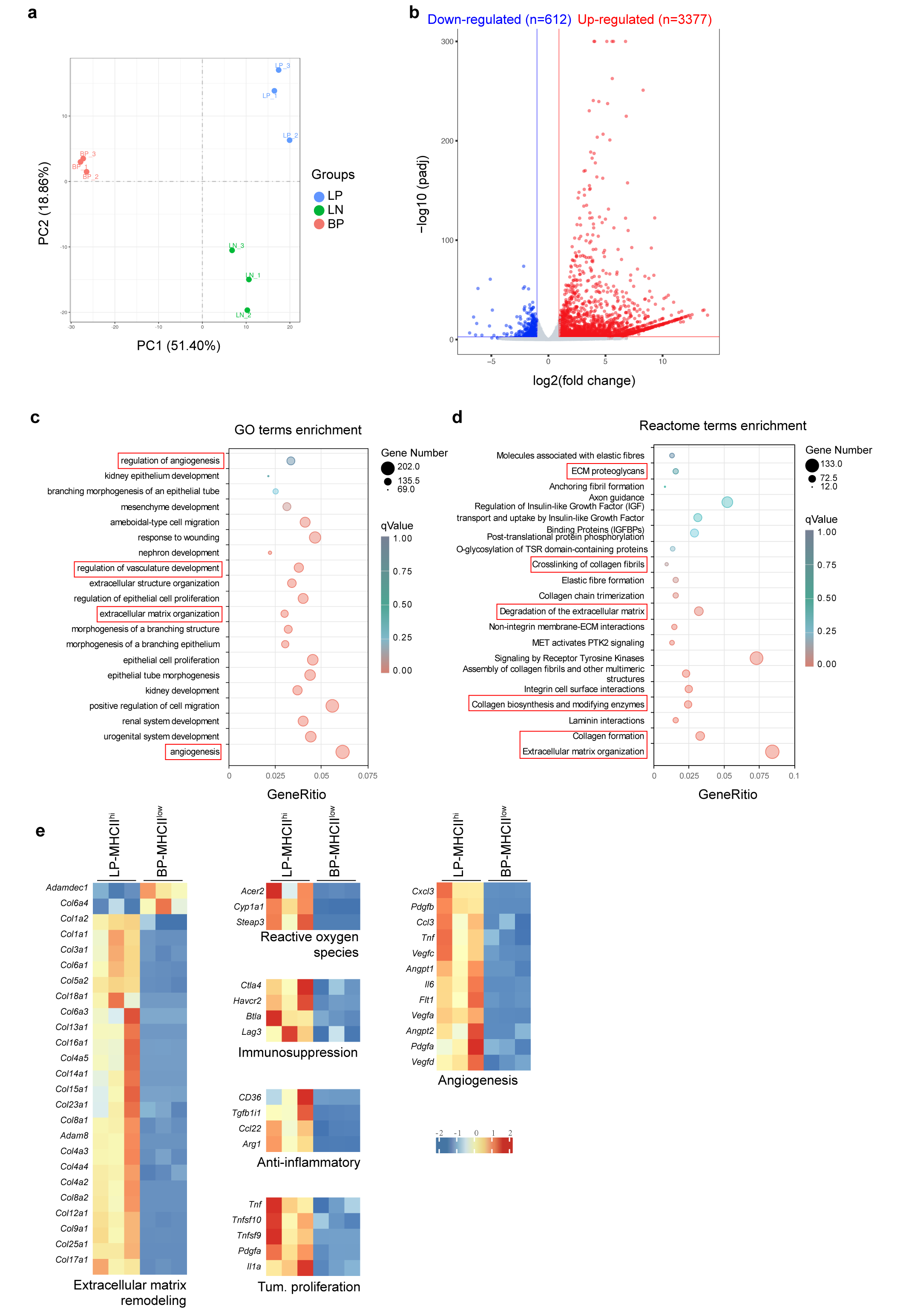


**Fig. S7 | Phenotyping of MHCII^hi^ neutrophils in the peripheral blood and lungs of chronically infected tumor-bearing mice. a.** Principal component analysis (PCA) of RNA-Seq in the MHCII^hi^ neutrophils of peripheral blood and MHCII^hi^ and MHCII^low^ neutrophils in the lungs. LP, MHCII^hi^ neutrophils derived from the lungs of mice; LN, MHCII^low^ neutrophils derived from the lungs of mice; BP, MHCII^hi^ neutrophils derived from the peripheral blood of mice. **b.** Volcano plot showing differential gene expression between the MHCII^hi^ neutrophils isolated from the blood and lungs of 4T1-bearing mice with chronic PAO1 infection. **c.** GO enrichment analysis of the differentially expressed genes between the MHCII^hi^ neutrophils in the peripheral blood and lungs of 4T1-bearing mice with chronic PAO1 infection. **d.** Reactome pathway analysis of the enriched genes identified from MHCII^hi^ neutrophils in the peripheral blood and lungs of 4T1-bearing mice with chronic PAO1 infection. **e.** Average expression levels of genes involved in extracellular matrix remodeling, reactive oxygen species, immunosuppression, anti-inflammation, angiogenesis, and tumor proliferation in MHCII^hi^ neutrophils isolated from the blood (BP-MHCII^hi^) and lungs (LP-MHCII^hi^) of 4T1-bearing mice with chronic infection.


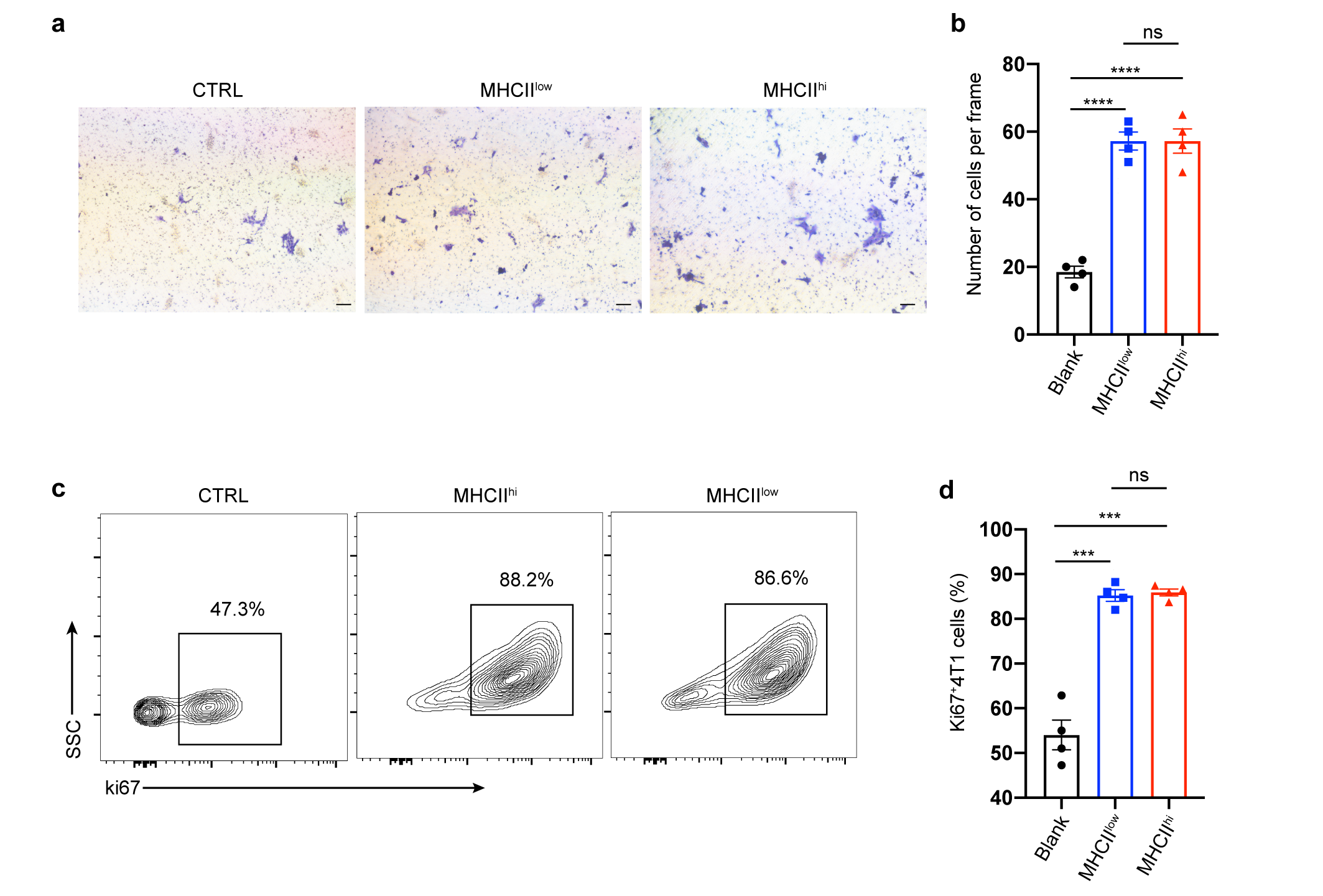


**Fig. S8 | Effects of MHCII^hi^ neutrophils on 4T1 tumor cell proliferation and invasion. a-b.** Representative pictures (**a**) display the effects of MHCII^hi^ and MHCII^low^ neutrophils on 4T1 cell invasion detected by Transwell assays. 4T1 cells were added to the upper chamber, MHCII^hi^ or MHCII^low^ neutrophils were added to the bottom chamber (8 μm pores), and the cells that migrated into the lower chamber were counted (**b**). n = 3; Scale bar, 200 μm. **c-d**. Flow cytometry plots (**c**) and percentage (**d**) of Ki67 expression in 4T1 cells. 4T1 cells were cocultured with either MHCII^hi^ or MHCII^low^ neutrophils for 24 h, stained with Ki67 and analyzed by flow cytometry. n = 4. Scale bar, 100 μm. All data are presented as the mean ± S.E.M. Statistical values were calculated using one-way ANOVA. ns, not significant; *****P* < 0.0001; ****P* < 0.001.


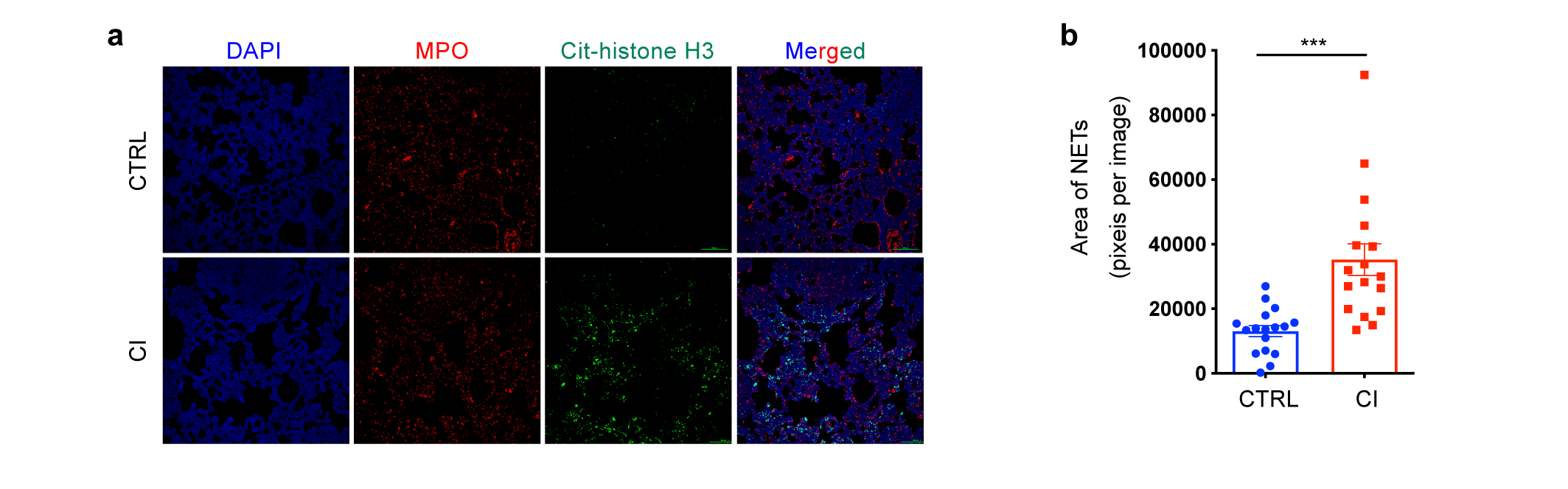


**Fig. S9 | *P. aeruginosa*-induced chronic infection promotes NET formation *in vivo.* a-b.** Representative images of immunofluorescence staining (**a**) and quantification (**b**) of NETs in the lungs of tumor-bearing mice with either PBS or PAO1 beads (chronic infection). Twenty fields of view were randomly imaged from 4 mice/group. Blue: DAPI; red: MPO; green: cit-H3. Scale bar, 100 μm. n = 4 mice/group. Scale bar, 100 μm. All data are presented as the mean ± S.E.M. Statistical values were calculated using an unpaired t test. ****P* < 0.001.


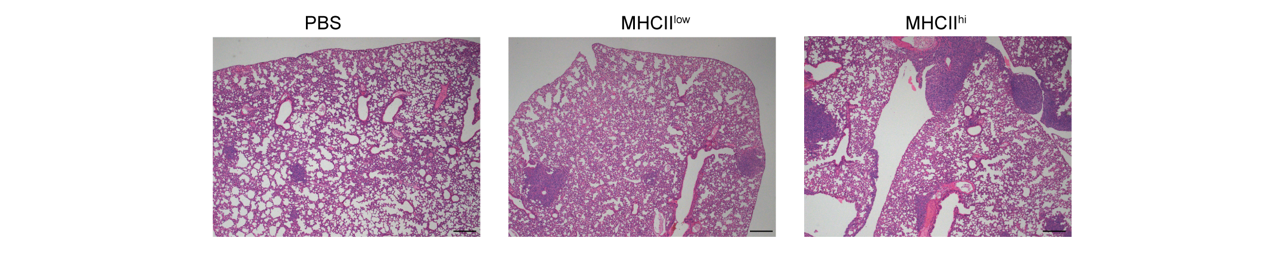


**Fig. S10 | MHCII^hi^ cells aggravate lung metastatic burden.** ﻿H&E staining of metastatic loci in the lungs of tumor-bearing mice infused with PBS, MHCII^hi^ and MHCII^low^ neutrophils. n = 4-7 mice/group. Scale bar, 100 μm


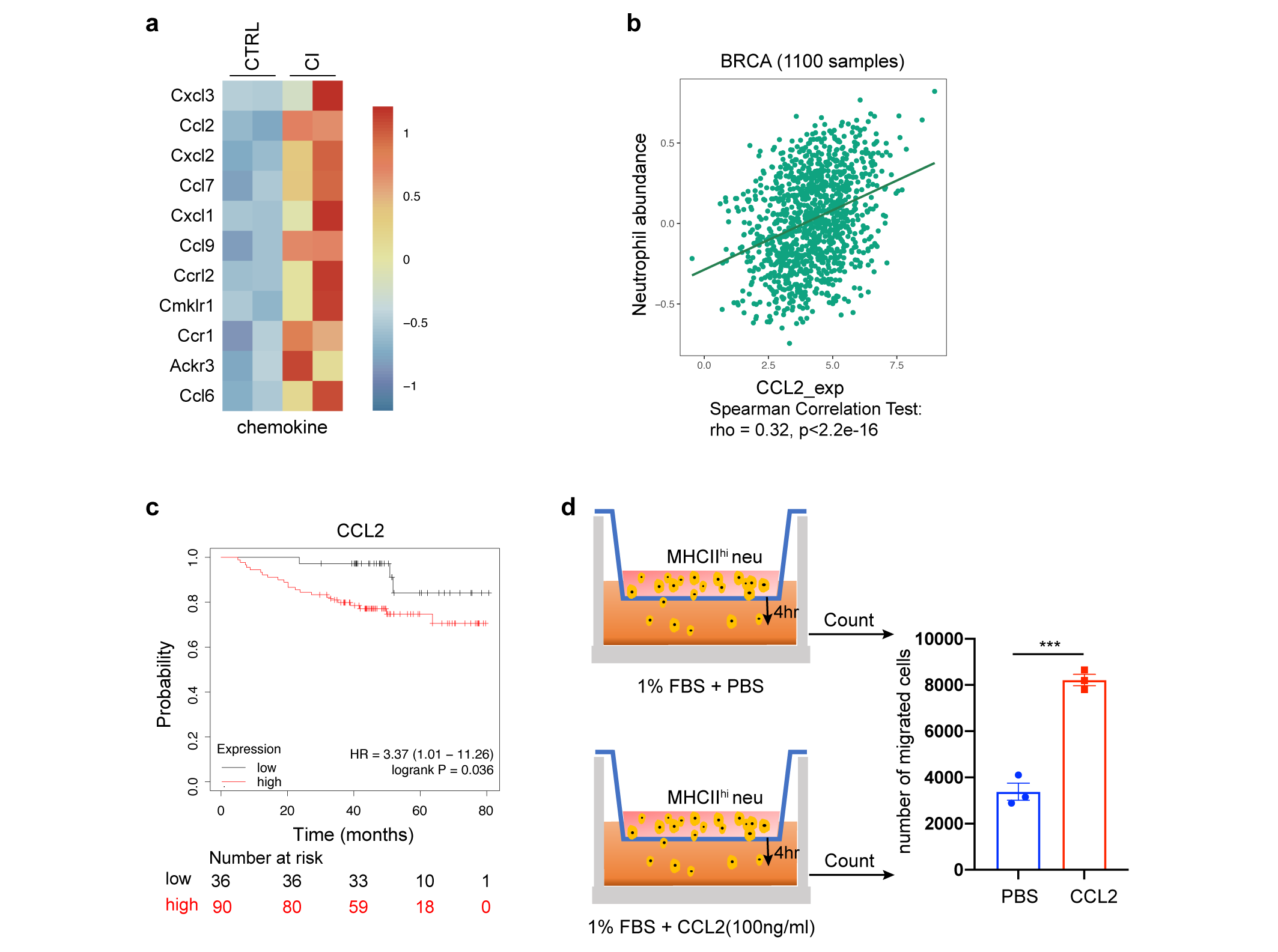


**Fig. S11 | Increased levels of CCL2 induced by chronic pulmonary infection recruit MHCII^hi^ cells into the lung. a.** A heatmap exploring the differences in chemokine gene expression in the lungs of tumor-free mice with or without chronic PAO1 infection. **b.** Correlation between Ccl2 expression and neutrophil abundance in breast cancer patients as examined by the TISIDB database. **c.** Survival plots of breast cancer patients (triple negative; n = 126) showing the correlation between poor prognosis and overexpression of Ccl2. Data were obtained from the *Kaplan‒Meier* *plotter* database. **d.** Experimental design of the Transwell migration assay. MHCII^hi^ neutrophils isolated from the blood of 4T1-bearing mice were added to the top chamber (1×10^5^ cells). The chemoattractant CCL2 (100 ng/ml) or control PBS was added to the bottom chamber. After incubation for 4 h, the cells harvested from the lower chamber were counted using a hemocytometer. All data are presented as the mean ± S.E.M. Statistical values were calculated using an unpaired t test. ****P* < 0.001.


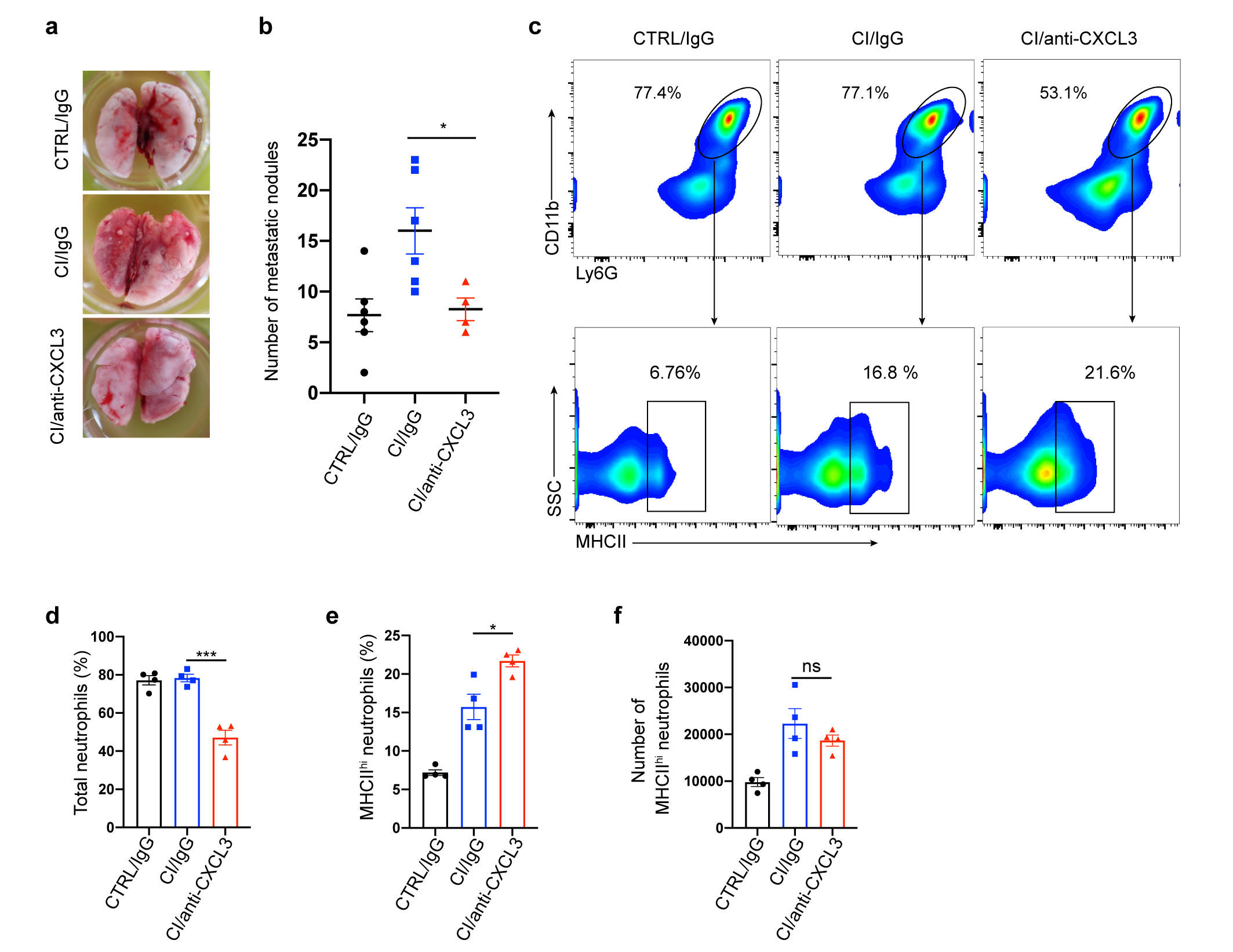


**Fig. S12 | CXCL3 neutralizing antibody suppresses breast cancer lung metastasis but does not specifically reduce MHCII^hi^ cell recruitment into the lung.** **a-b.** Bright-field imaging (**a**) and number of metastatic nodules (**b**) in the lungs of CTRL/IgG, CI/IgG, CI/CXCL3-block mice at the endpoint. After the establishment of 4T1-bearing mouse models with or without chronic PAO1 infection, the CXCL3-neutralizing antibody and isotype control were instilled into the lungs of mice. On Day 21, the mice were sacrificed, and the metastatic nodules on the lung were counted. n = 4-6 mice/group. **c-f.** Representative dot plots showing the change in the proportion of MHCII^hi^ neutrophils in the lung tissue of mice after neutralizing CXCL3 (**c**). The experimental setup is shown in **Fig. 6e**. The mice were sacrificed on Day 21, and the proportion of total neutrophils (**d**) or MHCII^hi^ neutrophils (**e**) and the number of MHCII^hi^ neutrophils (**f**) in the lungs of mice were determined by FACS analysis. n = 4-6 mice/group. All data are presented as the mean ± S.E.M. Statistical values were calculated using one-way ANOVA. ns, not significant; ****P* < 0.001; **P* <0.05.

**Table S1: Antibodies used for CyTOF stating**

| Mag-Tag | Antibodies | Clone | Vendor | Catalot |
| --- | --- | --- | --- | --- |
| 89Y | CD45 | 30-F11 | Biolegend | 103102 |
| 115In | CD3 | 145-2C11 | Biolegend | 100302 |
| 139la | ki67 | SolA15 | eBioscience | 14-5698-82 |
| 141Pr | CCR5/CD195 | C34-3448 | BD | 559921 |
| 142Nd | MHCII | Y3P | Bio-Xcell | BE0178 |
| 143Nd | TIM-3 | RMT3-23 | Biolegend | 119702 |
| 145Nd | CD163 | S15049I | Biolegend | 155302 |
| 146Nd | Ter119 | TER-119 | Biolegend | 116202 |
| 147Sm | Ly6G | 1A8 | Biolegend | 127602 |
| 148Nd | Ly6C | HK1.4 | Biolegend | 128002 |
| 149Sm | CD19 | 6D5 | Biolegend | 115502 |
| 150Nd | CD40 | HM40-3 | Biolegend | 102902 |
| 151Eu | B220 | RA3-6B2 | Biolegend | 103202 |
| 152Sm | CD11c | N418 | Biolegend | 117302 |
| 153Eu | CD44 | IM7 | Biolegend | 103002 |
| 154Sm | CCR3 | J073E5 | Biolegend | 144502 |
| 155Gd | Lag3 | C9B7W | Biolegend | 125202 |
| 156Gd | CCR4/CD194 | 2G12 | Biolegend | 131202 |
| 157Gd | NKp46 | 29A1.4 | Biolegend | 137602 |
| 158Gd | TCRgd | UC7-13D5 | Biolegend | 107502 |
| 159Tb | F4/80 | C1:A3-1 | BioRAD | MCA497G |
| 160Gd | TCRb | H57-597 | Biolegend | 109202 |
| 161Dy | TIGIT | 2190A | RD | MAB72671 |
| 162Dy | CD103 | 2E7 | Biolegend | 121402 |
| 163Dy | CD25 | 3C7 | Biolegend | 101902 |
| 164Dy | CCR7 | 4B12 | Biolegend | 120101 |
| 165Ho | CD64 | X54-5/7.1 | Biolegend | 139302 |
| 166Er | Arg1 | E-2 | Santa Cruz Biotechnology | sc-271430 |
| 167Er | CD49b | DX5 | Biolegend | 108902 |
| 168Er | FoxP3 | FJK-16s | eBioscience | 14-5773-82 |
| 169Tm | CD62L | MEL-14 | Biolegend | 104402 |
| 170Er | iNOS | CXNFT | eBioscience | 14-5920-82 |
| 171Yb | Siglec-H | 440c | Gene Tex | GTX14268 |
| 172Yb | PD-1 | 29F.1A12 | Biolegend | 135202 |
| 173Yb | CD172a | P84 | Biolegend | 144002 |
| 174Yb | CCR2 | 475301 | RD | MAB55381-100 |
| 175Lu | Siglec-F | E50-2440 | BD | 552125 |
| 176Yb | MerTK | 2B10C42 | Biolegend | 151502 |
| 197Au | CD4 | RM4-5 | Biolegend | 100520 |
| 198Pt | CD8 | 53-6.7 | Biolegend | 100716/100746 |
| 209Bi | CD11b | M1/70 | RD | FAB1124F-025/FAB1124F-100 |

**Table S2: Demographic Characteristics of the patients with breast cancer lung metastasis**

| ID | Age  years | WHO   Grade | Metastasis  loci | Estrogen receptor | Progesterone receptor | HER2  status | Proliferation index  (Ki-67) | Molecular classes |
| --- | --- | --- | --- | --- | --- | --- | --- | --- |
| 1 | 19 | 7 scores, 2 | lung | positive | negative | 2+ | 10% | Luminal A |
| 2 | 46 | 7 scores, 2 | lung | positive | negative | negative | 70% | Luminal B |
| 3 | 54 | uncertain | lung | negative | positive | negative | 60% | Luminal B |
| 4 | 66 | 5 scores, 1 | lung | positive | negative | negative | 50% | Luminal B |
| 5 | 55 | 6 scores, 2 | lung | positive | negative | 2+ | 2% | Luminal B |
| 6 | 62 | 7 scores, 2 | lung | positive | positive | 2+ | 20% | Luminal B |
| 7 | 46 | 7 scores, 2 | lung | negative | negative | 1+ | 70% | Basal-like |
| 8 | 47 | 6 scores, 2 | lung | positive | positive | 3+ | 80% | Luminal B |
| 9 | 45 | 7 scores, 2 | lung | positive | negative | 3+ | 70% | Luminal B |
| 10 | 59 | 7 scores, 2 | lung | positive | positive | 3+ | 30% | Luminal B |
| 11 | 34 | 6 scores, 2 | lung | negative | negative | negative | 10% | Basal-like |
